# On Neotropical Merophysiinae with descriptions of a new genus and new species (Coleoptera, Endomychidae)

**DOI:** 10.3897/zookeys.736.21628

**Published:** 2018-02-08

**Authors:** Emmanuel Arriaga-Varela, Wioletta Tomaszewska, Lizhi Huo, Matthias Seidel

**Affiliations:** 1 Department of Zoology, Faculty of Science, Charles University, Prague, Viničná 7, CZ-12843, Prague, Czech Republic; 2 Museum and Institute of Zoology PAS; Wilcza 64, 00-679 Warszawa, Poland; 3 Engineering Research Centre of Biological Control, Ministry of Education, South China Agricultural University, Guangzhou, 510642 China

**Keywords:** Coccinelloidea, Entomology, *Lycoperdinella*, new genus, new species, *Rueckeria*, taxonomy

## Abstract

Intensive survey of museum collections and new field collecting resulted in discovery of six new, closely related species of the Neotropical Merophysiinae. A new species of the genus *Lycoperdinella* Champion, *L. boliviensis* **sp. n**., from Bolivia and Brazil, and five new species from Mexico for which a new genus is proposed here as *Rueckeria* **gen. n**.: *R. inecol* (type species), *R. nigrileonis*, *R. ocelotl*, *R. puma*, *R. skelleyi* **spp. n**., have been discovered. *Lycoperdinella*, *Rueckeria* **gen. n**., *L. subcaeca* Champion and all new species are diagnosed, described, and illustrated. Keys to the species of *Lycoperdinella* and *Rueckeria* and a distribution map are provided. A lectotype of *Lycoperdinella subcaeca* Champion, 1913 is designated. Molecular barcodes of three new species of *Rueckeria* are provided in order to help with the identification of these taxa.

## Introduction

Merophysiinae is a subfamily of Endomychidae, a moderately large family of mycophagous beetles distributed worldwide but with highest diversity in the tropical regions (Shockley et al. 2009a, b). The family, historically classified in the superfamily Cucujoidea (e.g., Lawrence and Newton 1995, Tomaszewska 2000, 2010, Shockley et al. 2009a) has recently been redefined by the exclusion of former subfamilies Eupsilobiinae, Mycetaeinae, and Anamorphinae which have been elevated to the level of independent families, all classified now in the superfamily Coccinelloidea (Robertson et al. 2015).

Merophysiinae (= Holoparamecinae) (Tomaszewska 2000, 2005) comprise 12 genera and more than 100 species (Shockley et al. 2009a) with most species diversity in the Old World. Merophysiines are usually small in size, ranging from one to three millimeters. The group is well supported morphologically by having the tentorium without a corpotentorium, labial palpomere 2 oval and inflated, and larval head lacking stemmata (Tomaszewska 2000, 2005). Molecular study (Robertson et al. 2015) also supports monophyly of this subfamily.

The Neotropical region is inhabited by five merophysiine genera. *Holoparamecus* Curtis, 1833, including numerous species distributed worldwide, and four genera endemic to the Neotropics: *Colovocerida* Belon, 1884, *Lycoperdinella* Champion, 1913, *Pseudevolocera* Champion, 1913 and *Pseudoparamecus* Brèthes, 1922. However, apart from the often studied and well known *Holoparamecus*, and carefully studied (during preparation of this paper) type material of monotypic *Lycoperdinella*, the remaining three genera are rather enigmatic. *Pseudevolocera* was described as having a 10-segmented antenna with 3-segmented club, prosternum having large fossae for the reception of antennal club, pronotum with basal groove but without foveae, tarsi 3-segmented, metaventral postcoxal lines, and weak abdominal lines present. Champion (1913) himself suggested close relationships between *Pseudevolocera* and *Evolocera* (which is a member of the family Eupsilobiidae). *Colovocerida*, as described by Belon (1884), has an 11-segmented antenna with 2-segmented club, 4-segmented tarsi with tarsomere 3 shortened, and abdominal ventrite 1 with postcoxal lines. Finally, *Pseudoparamecus*, according to Brèthes (1922) has a 10-segmented antenna with 3-segmented club, front and hind coxae subcontiguous, and mid coxae contiguous. From the original descriptions of these genera (diagnostic combination of characters) it seems that they doubtedly belong to Merophysiinae or even to any other Endomychidae subfamily. Their taxonomic position needs further study.

In addition to the information about the introduced species of *Holoparamecus*, which probably feed on the molds of stored grains (Shockley et al. 2009b), almost nothing is known about the biology of the neotropical species of Merophysiinae. The most noteworthy exception is *Holoparamecus gabrielae* Rücker which was collected in a pile of bat guano in a cave of central Veracruz, Mexico (Rücker 2003). The gut content of a new species of *Lycoperdinella* described here shows fragments of phragmospores, suggesting that it probably feeds on Ascomycetes fungi.

A recent survey of the Endomychidae material from museum collections (National Museum in Prague; The Natural History Museum in London; Florida State Collection of Arthropods in Gainesville, FL; Museum and Institute of Zoology in Warsaw) carried out by the authors, and recent field collecting in Mexico, carried out by EA-V and MS, revealed the new discoveries reported here.

Six new species of Merophysiinae from Central and South America have been discovered and are described in this paper. A new species of *Lycoperdinella* from Bolivia and Brazil, and five new species from Mexico for which a new genus is proposed here as *Rueckeria* gen. n.

The genus *Lycoperdinella* was established by Champion (1913) for *Lycoperdinella subcaeca*, described originally from Guatemala (Champion 1913). The discovery of the new species from Bolivia and Brazil extends considerably the distribution range of the genus southward in the Neotropical region.

## Material and methods

Acronyms for the depositories of specimens are:

**BMNH** The Natural History Museum, London, United Kingdom;

**CNIN** National Collection of Insects, UNAM, Mexico City, Mexico;

**CZUG** Center of Zoological Studies, University of Guadalajara, Zapopan, Mexico;

**FSCA** Florida State Collection of Arthropods, Gainesville, FL, USA;

**IEXA** Institute of Ecology, Xalapa, Mexico;

**MIZ** Museum and Institute of Zoology PAS, Warszawa, Poland;

**MNKM** Museo de Historia Natural Noel Kempff Mercado, Santa Cruz de la Sierra, Bolivia;

**NMPC** National Museum, Prague, Czech Republic;

**USNM** United States National Museum of Natural History, Washington D.C., USA.

Genitalia of both sexes, if available, were dissected, cleared in 10% KOH solution and rinsed with distilled water, then transferred to glycerol and examined on slides. After examination the genitalia were transferred to microvials and pinned beneath the specimens. Measurements were made using an ocular micrometer attached to an Olympus (SZX 16) dissecting microscope. The following measurements were made and are used in descriptions: TL – total length, from apical margin of clypeus to apex of elytra; PL – pronotal length, from the middle of anterior margin to margin of basal foramen; PW – pronotal width at widest part; EL – elytral length across sutural line including scutellum; EW – elytral width across both elytra at the widest part. Habitus photographs were taken using a Canon EOS 550D digital camera with attached Canon MP-E65mm f/2.8 1–5× macro lens, and subsequently modified in Adobe Photoshop CS5. The photographs of the genitalia and the other disarticulated morphological structures were taken using a Canon EOS 1100D digital camera attached to an Olympus BX41 compound microscope and subsequently combined using Helicon Focus software. Scanning electron micrographs were taken using a Hitachi S-3700N environmental electron microscope at the Department of Palaeontology, National Museum in Prague and a HITACHI S-3400N microscope (www.hitachi.com) in the Electron Microscopy Laboratory at the Museum and Institute of Zoology, Polish Academy of Sciences in Warsaw.

The species descriptions of *Lycoperdinella* start from the type species and follow by a new species; the species of *Rueckeria* gen. n., are ordered alphabetically.

The beetle-specific terminology and numbering of body parts follow Lawrence et al. (2011). Classification follow Tomaszewska (2010).

**DNA barcoding.** Most of the examined specimens were collected during a recent expedition to Mexico. Samples were preserved in 96 % alcohol and stored at -20 °C. DNA was extracted from complete specimens using a Qiagen Blood and Tissue DNA extraction kit following the manufacturer's, instructions. The highly variable 5’ region of the mitochondrial cytochrome c oxidase subunit I gene (COI) was amplified using LCO1490 (5’-GGTCAACAAATCATAAAGATATTGG-3’) and HCO2198 (5’-TAAACTTCAGGGTGACCAAAAAATCA-3’) primers (Folmer et al. 1994). Each 10 µl PCR reaction contained 6.7 µl H_2_O, 0.4 µl of MgCl_2_ (25 mM), 0.2 µl of dNTPs (10 mM), 0.3 µl of each forward and reverse primer (10 µM), 0.1 µl of Taq polymerase (5 u/µl), 1.0 µl of 10x Taq buffer, and 1.0 µl of DNA template. The PCR conditions consisted of 3 min at 94 °C + 35 cycles of 30 s at 94 °C, 45 s at 48 °C and 1 min at 72 °C + 8 min at 72 °C. 5 µl of each PCR product were purified by adding 0.5 µl (20 u) Exonuclease I (Exo1) and 1µl (1 u) Thermosensitive Alkaline Phosphatase (FastAP) (Thermo Fisher Scientific) and incubating the mixture for 15 min at 37 °C, followed by 15 min at 80 °C. Sequences were edited with Geneious 9. We did not attempt DNA extraction and sequencing of museum specimens, that is why only sequences for three species of *Rueckeria* gen. n. are presented.

## Taxonomy

### Genus and species descriptions

#### Lycoperdinella

Taxon classificationAnimaliaColeopteraEndomychidae

Champion

**Figure 1.**
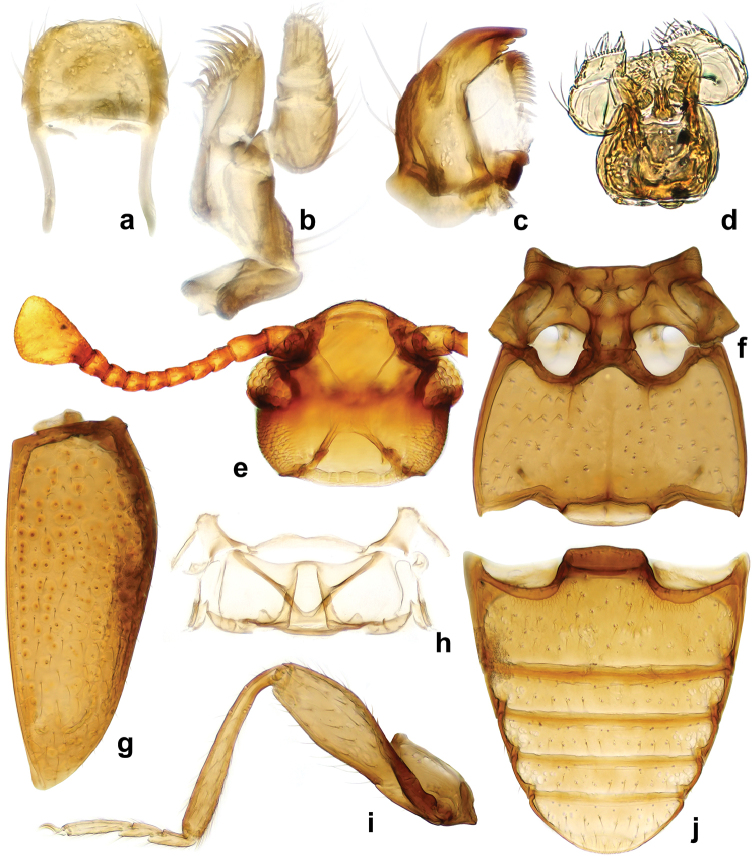
*Lycoperdinella boliviensis* sp. n. **a** labrum **b** maxilla **c** mandible **d** labium **e** ventral view of head with buccal parts removed **f** meso- and metaventrite **g** right elytron **h** metanotum **i** hind leg **j** abdominal ventrites.

**Figure 2.**
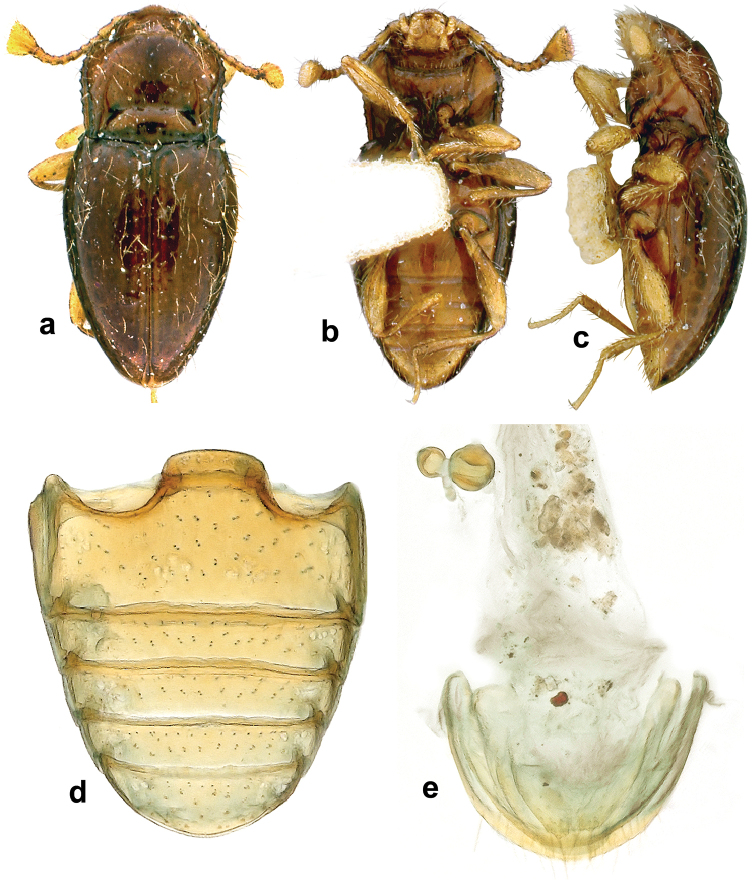
*Lycoperdinella subcaeca* Champion, paralectotype **a** dorsal habitus **b** ventral habitus **c** lateral habitus **d** abdominal ventrites **e** female genitalia.

**Figure 3.**
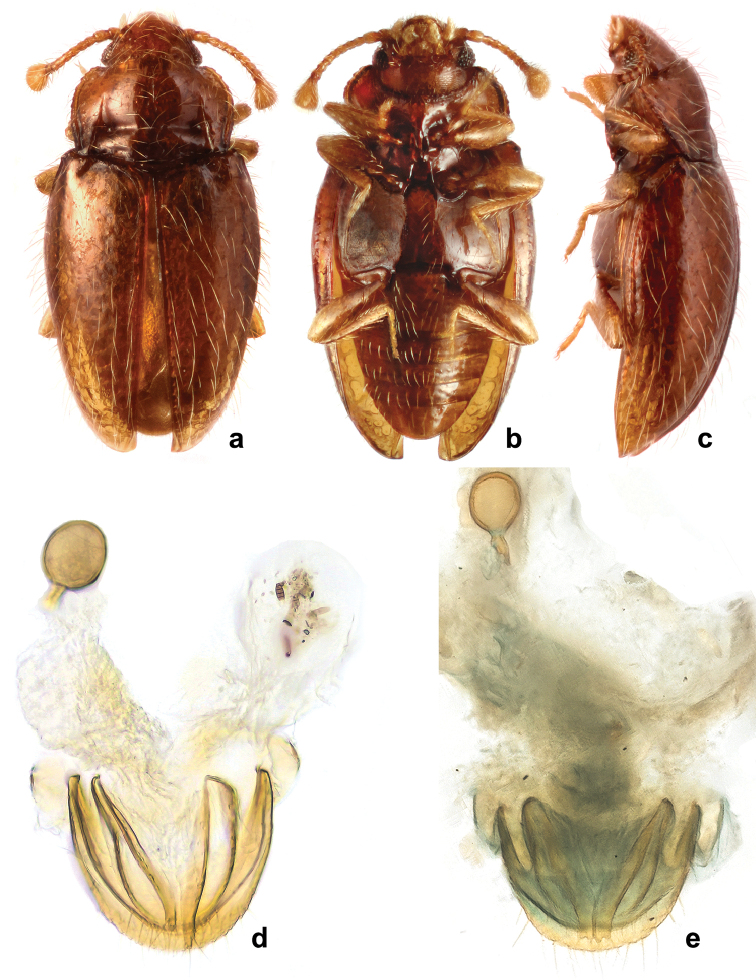
*Lycoperdinella boliviensis* sp. n. **a** dorsal habitus **b** ventral habitus **c** lateral habitus **d** female genitalia of Bolivian paratype **e** female genitalia of Brazilian paratype.

**Figure 4.**
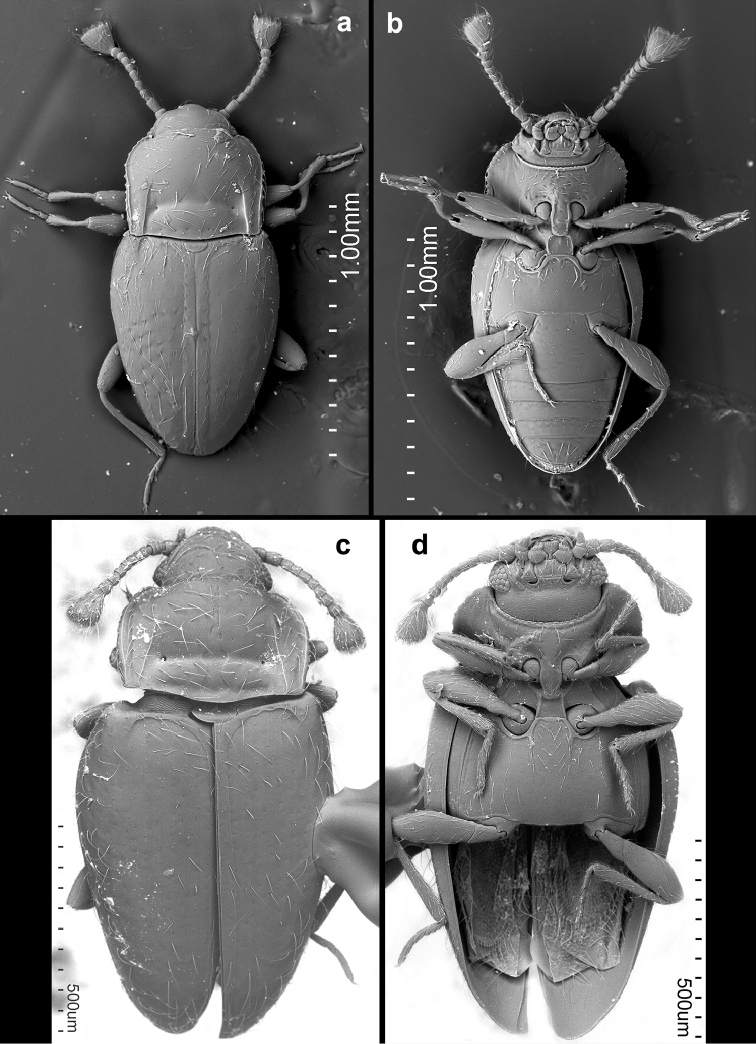
*Lycoperdinella* species **a–b** *Lycoperdinella subcaeca* Champion **c–d** *Lycoperdinella boliviensis* sp. n. **a** dorsal view **b** ventral view **c** dorsal view **d** ventral view.

**Figure 5.**
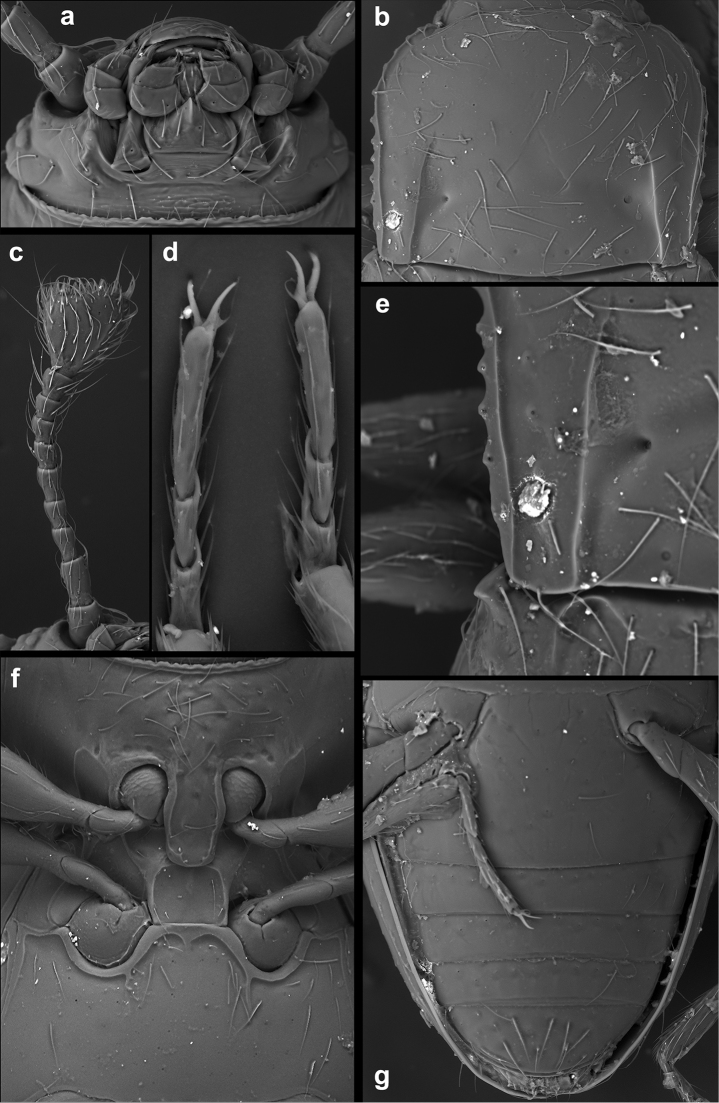
*Lycoperdinella subcaeca* Champion, lectotype **a** ventral view of head **b** pronotum **c** antenna **d** meso- and metatarsus **e** postero-lateral corner of pronotum and antero-lateral corner of elytron **f** pro-, meso- and metaventrite **g** abdominal ventrites.

**Figure 6.**
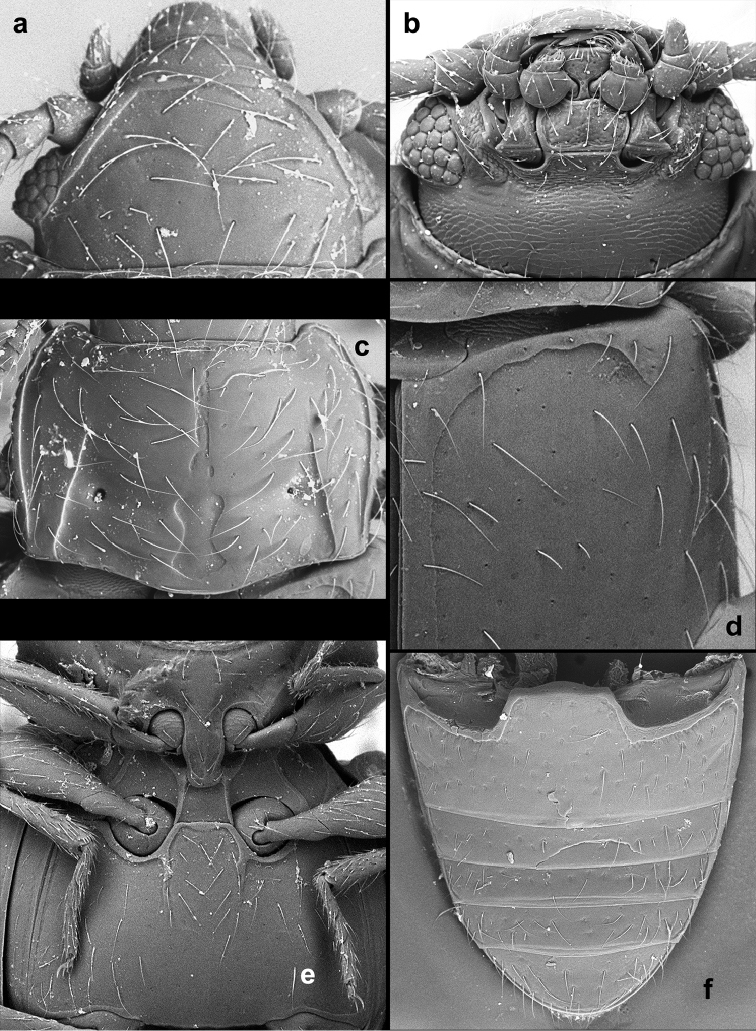
*Lycoperdinella boliviensis* sp. n. **a** dorsal view of head **b** ventral view of head **c** pronotum **d** antero-lateral corner of elytron **e** pro-, meso- and metaventrite **f** abdominal ventrites.

Figs 1 , 2 , 3 , 4 , 5 , 6 , 21

Lycoperdinella Champion, 1913: 114. Type species: Lycoperdinella subcaeca Champion, 1913 (by monotypy). – Shockley et al. 2009a: 69; Robertson et al. 2015: 766.

##### Diagnosis.

*Lycoperdinella* can be distinguished from other Neotropical Merophysiinae by the following combination of characters: antenna 10-segmented with 1-segmented club (Fig. 1g); antennal grooves on head absent or indistinct (Figs 5a, 6a); pronotum with lateral margins narrowly bordered and distinctly crenulate (Figs 5b, e, 6c); metaventrite without postcoxal lines (Fig. 1f); abdominal ventrite 1 without postcoxal lines (Figs 1j, 2d). *Lycoperdinella* is most similar to *Rueckeria* gen. n. and *Holoparamecus* but from *Rueckeria* it can be differentiated by the lateral margins of the pronotum being coarsely crenulate (at most weakly crenulate in *Rueckeria*), elytron with anterolateral corner with a hooked tooth (Figs 1g, 5e, 6d) (anterolateral corners of elytra simple in *Rueckeria*), postcoxal lines absent on abdominal ventrite 1 (postcoxal lines present in *Rueckeria*), shorter and stouter antennae (Fig. 1e), the body covered with much longer, suberect setae (Fig. 6d) (short setae in *Rueckeria*) and the hind wings present in one of two species. From *Holoparamecus*, *Lycoperdinella* can be separated by having lateral margins of the pronotum bordered (smooth in *Holoparamecus*), pronotum narrowest near base (Figs 5b, 6c) but not distinctly constricted basally as in *Holoparamecus*, antenna with 1-segmented club (2-segmented club present in *Holoparamecus*).

##### Redescription.

Length 1.3–1.4 mm. Body elongate, approx. 2.2 times longer than wide, weakly convex, approx. 3.3 times as long as high; shiny, smooth, covered with sparse and long pale setae. Color light brown.

Head (Figs 1e, 5a, 6a, b) deeply retracted in prothorax, slightly wider than long; sparsely and moderately densely punctate. Gular sutures subparallel, widely separated. Eyes very small, oval, coarsely faceted, composed of six or 36 facets (as based on the studied specimens). Antennal sockets concealed by sides of frons, not visible from above; antennal grooves absent. Antenna comparatively short (Figs 1e, 5c), almost reaching base of pronotum, composed of ten antennomeres with club formed by terminal antennomere which is large, inflated, subtriangular, truncate apically. Frontoclypeal suture weakly arcuate (Fig. 6a). Clypeus transverse, flat, convergent anteriorly. Labrum (Fig. 1a) subquadrate, with rounded anterolateral corners, truncate apically, with submembranous apex, punctate, covered with sparse, long setae; tormae with mesal arms recurved posteriorly; labral rods absent. Mandible (Fig. 1c) with two apical teeth, and with four smaller subapical teeth getting subsequently smaller posteriorly; prostheca covered with digitiform setae on anterior third getting thinner and shorter toward posterior 2/3; mola large, sclerotized; submola small, membranous. Maxilla (Fig. 1b) with palpomere 1 and 3 very short; palpomere 2 large and swollen, almost twice as long as palpomere 3; terminal palpomere approx. 1.5 times as long as 2 and 3 palpomeres combined, narrow, tapering, apex obliquely truncate to weakly rounded; galea moderately broad, twice as broad as lacinia, with long, broad, apical spine-like setae; lacinia elongate, with spine-like setae on apical half. Labium (Fig. 1d) with palpomere 1 very small; palpomere 2 largest, oval, inflated; terminal palpomere transverse, truncate at apex. Mentum subquadrate, with produced anterior angles; finely punctate, glabrous. Prementum subquadrate, sclerotized, with apically expanded membranous ligula. Tentorium (Fig. 1e) with anterior arms fused medially and widely divergent anteriorly; corpotentorium absent.

Prothorax. Pronotum (Figs 5b, e, 6c) weakly transverse, widest at anterior 1/4 to 1/3 then roundly narrowing basally; pronotal surface finely and sparsely punctate; lateral edges moderately widely bordered, strongly crenulate; anterior margin curved with slightly projected, rounded angles; posterior angles almost right-angled. Pronotal disc weakly convex. Pronotal base with an impression composed of two longitudinal sharply defined sulci, slightly convergent anteriorly, almost reaching apical fourth, and a pair of deep transverse linear depressions/sulci. Anterior transverse sulcus flanked by one small deep fovea on each side; area between transverse sulci convex; basal sulcus not reaching lateral sulci, with or without large foveate punctures. Prosternal process (Figs 5f, 6e) broad, approx. as wide as coxal diameter, with raised margins; extending posteriorly beyond front coxae. Procoxa circular in outline, its cavity externally open behind, internally closed; trochantin concealed (Figs 5f, 6e).

Meso- and metathorax. Mesonotum sclerotized; scutellar shield small, strongly transverse, widely rounded apically, partially covered by base of pronotum. Mesoventrite (Fig. 1f) carinate at middle; intercoxal process moderately elongate, rather broadly separates mesocoxae (slightly narrower or slightly wider than coxal diameter) reaching half of their length. Mesoventrite (Figs 1f, 5f, 6e) fused with mesanepisternum (trace of suture visible). Mesocoxa weakly oval in outline, its cavity narrowly closed outwardly by sterna; trochantin concealed. Meso-metaventral junction of straight-line type, without internal knobs. Elytron (Figs 4a, c, 6d) elongate, convex, irregularly and moderately finely punctate, with a hooked tooth on anterolateral corner, epipleuron narrow, incomplete at apex. Sutural stria sharply defined, complete, widest at mid length, then weakly converging towards elytral apex. Metaventrite (Fig. 1f) transverse, twice as long as mesoventrite, weakly convex; with postcoxal longitudinal ridges nearly reaching anterior 2/5; anterior margin rather thick; discrimen very short. Metanotum (Fig. 1h) sclerotized. Metacoxae transversely oval, widely separated. Metendosternite with very short stalk and moderately widely separated anterior tendons. Hind wings absent or present and well developed, longer than elytra, with anal, medial and radial fields elongate and narrow, apical field enlarged; margins surrounded with long hairs.

Legs. Trochanter moderately elongate (Fig. 1i); trochantero-femoral attachment oblique. Femur widest near middle of its length, more than twice as wide as tibia, sparsely setose; tibia and tarsus covered with long, dense setae. Tibia narrow, straight or slightly bent inwards, continuously weakly widened distally or with abruptly wider part at distal third, without apical spurs. Tarsal formula 3-3-3 in both sexes (Fig. 5d): tarsomere 1 approx. 1.5 times longer than 2; tarsomere 3 slightly longer than remaining tarsomeres combined. Claws simple. Empodium very small.

Abdomen (Figs 1j, 5g, 6f) with five freely articulated ventrites; ventrite 1 slightly longer than three following ventrites together, without postcoxal lines; ventrites 2–4 almost equal in length; ventrite 5 long, acuminate, nearly as long as ventrites 3–4 together.

Male not known.

Female genitalia (Figs 2e, 3d, e). Ovipositor weakly sclerotized, with coxites elongate; styli vestigial, placed apically. Spermatheca moderately large, submembranous, more or less distinctly two-chambered with at least one chamber rounded; sperm duct moderately long, slender; accessory gland small membranous, elongate or oval.

##### Distribution.

Central and South America: Bolivia, Brazil, Costa Rica, Guatemala (Fig. 21).

#### Lycoperdinella subcaeca

Taxon classificationAnimaliaColeopteraEndomychidae

Champion

Figs 2 , 4a–b , 5 , 21

Lycoperdinella subcaeca Champion, 1913: 115. Type locality: Guatemala. – Shockley et al. 2009a: 69; Robertson et al. 2015: 766.

##### Differential diagnosis.

*Lycoperdinella subcaeca* is similar to *L. boliviensis* in its body shape, color and vestiture, however *L. subcaeca* can be separated from that species by having the pronotum more elongate (0.80 times as long as broad), eyes reduced to six facets only (in both type specimens studied), mentum somewhat pentagonal (sharply produced anteriorly in the middle of apical margin), the abdominal ventrite 1 longer than the mesoventrite and the hind wings absent.

##### Redescription.

Length 1.39 mm, width 0.66 mm, height 0.47 mm; body elongate-oval, moderately convex, 2.11 times as long as wide, 2.96 times as long as high (Figs 2a–c, 4a, b). Surfaces shiny; sparsely covered with long, decumbent, golden setae. Color homogeneously reddish brown.

Head with interocular distance 0.83 times as wide as head including eyes. Eyes very small, composed of six facets (Fig. 5a). Antenna rather short and slender (Fig. 5c), 0.83 times as long as head and pronotum combined; scape 1.52 times longer than wide, 1.09 times as long as pedicel; pedicel 1.88 times longer than wide; antennomere 3, 1.50 times longer than wide, 0.66 times as long as pedicel; antennomeres 4–8 getting very gradually shorter and wider towards antennomere 9, which is 0.98 times wider than long and 0.64 times as long as pedicel; terminal antennomere inflated, asymmetrical, 2.03 times as long at longer margin than pedicel, its longest margin 1.30 times longer than shorter lateral one and 1.18 times as long as apical margin; apical margin truncate. Mentum subquadrate with lateral margins weakly rounded and anterior margin sharply produced anteriorly at mid-line (Fig. 5a).

Pronotum weakly transverse (Fig. 5b), 0.80 times as long as wide, 1.50 times wider than head, 1.06 times wider at widest part than at base, widest at about anterior third, weakly convex; front angles very weakly produced, rounded, lateral margins almost rounded in anterior third, then converging to posterior angles, comparatively widely bordered with edges distinctly crenulate; hind angles weakly obtuse, rounded at tips. Posterior half of disc with a vaguely defined triangular impression. Longitudinal sulci distinctly convergent anteriorly, extending from base to almost half length of pronotum; well defined transverse sulcus connecting deep pores and weakly marked basal transverse depression provided with large punctures (Fig. 5e); area between transverse sulci/depressions weakly convex; posterior margin weakly lobed at mid-line. Prosternal process widely separates front coxae, widest at mid length.

Elytra 0.94 mm long, 1.42 times longer than wide; 2.19 times as long as and 1.22 times as wide as pronotum; widest at basal fourth then continuously distinctly converging to rounded apex; with hooked tooth present anterolateral corner. Hind wings absent.

Legs moderately long. Femora very narrow at base, strongly widened at apical half. Tibiae narrow, straight, continuously widened towards their apices. Metatibia very narrow, 0.34 times as long as elytra. Metatarsus moderately long, 0.6 times as long as metatibia.

Abdomen (Figs 2d, 5g) with ventrite 1 slightly shorter than metaventrite and as long as three following ventrites combined. Ventrite 5 arcuate at apex.

Female genitalia (Fig. 2e) with narrow coxites rounded at their apices; spermatheca distinctly two-chambered, chambers rounded; accessory gland small, elongate.

Male unknown.

##### Type material.

***Lectotype*** of *Lycoperdinella subcaeca* Champion, female, **GUATEMALA**, “Livingston, 65, Guat./ H.S. Barber Collector/ U.S. Nat. Mus. 1913–253, det. Champion/ Lycoperdinella subcaeca Ch./ Co-type” (BMNH). ***Paralectotype*** of *Lycoperdinella subcaeca* Champion, Guatemala, “Livingston, 65, Guat./ Barber & Schwarz Coll./ Type no. 21530, USNM/ Lycoperdinella subcaeca Ch., type” (USNM) [examined on photos].

##### Distribution.

Central America: Guatemala (Fig. 21).

##### Comments.

This species was listed by Shockley et al. (2009a) as present also in Costa Rica. However, due to a lack of any specimens accessible for examination from this country during our extensive study, a question mark is added in the distribution map for this species in Costa Rica.

Champion (1913) clearly indicated that his original description of *L. subcaeca* was based on two specimens from Guatemala. One left in ‘U.S. Nat. Mus.’ and the second specimen was ‘presented to the British Museum’. As the lectotype we have chosen and designated a specimen available for direct study (borrowed to WT from the NHM). The syntype from USNM, examined on photos becomes a paralectotype.

#### Lycoperdinella boliviensis sp. n.

Taxon classificationAnimaliaColeopteraEndomychidae

http://zoobank.org/305A07DA-3A5B-461B-A213-600667D60BD4

Figs 1 , 3 , 4c–d , 6 , 21

##### Etymology.

The name of this species is derived from the country of origin of the holotype.

##### Differential diagnosis.

*Lycoperdinella boliviensis* closely resembles *L. subcaeca* in its overall body shape, color and vestiture, but can be separated from *L. subcaeca* by having the pronotum more transverse (0.68–0.70 times as long as broad), eyes composed of 36 facets (based on studied specimens), mentum subrectangular with its anterior margin weakly arcuate, and the abdominal ventrite 1 shorter than the mesoventrite, and by the presence of well-developed hind wings.

##### Description.

Length 1.30–1.40 mm, width 0.60 mm, height 0.43 mm; body elongate-oval, moderately convex, 2.25 times as long as wide, 2.85 times as long as high (Fig. 3a–c). Surfaces shiny; sparsely covered with long, decumbent, golden setae. Color homogeneously reddish brown.

Head with interocular distance 0.75 times as wide as head including eyes (Fig. 6a, b). Eyes small, composed of 36 facets. Antenna rather short and slender (Fig. 1e), 0.72 times as long as head and pronotum combined; scape 1.33 times longer than wide, 1.15 times as long as pedicel; pedicel 1.15 times longer than wide; third antennomere 1.15 times longer than wide, 0.65 times as long as pedicel; antennomeres 4–8 getting very gradually shorter and wider towards antennomere 9 which is 1.28 times wider than long and 0.6 times as long as pedicel; terminal antennomere inflated, asymmetrical, 2.30 times as long at longer margin than pedicel, its longest margin 1.16 times longer than shorter lateral one, and 1.16 as long as apical margin, apical margin truncate. Mentum subquadrate, with lateral margins weakly rounded and anterior margin slightly arcuate (Fig. 6b).

Pronotum weakly transverse (Fig. 6c), 0.68–0.70 times as long as wide, 1.53–1.57 times wider than head, about 1.05 times wider at widest part than at base, widest at about mid length, weakly convex; front angles weakly produced, rounded, lateral margins almost continuously rounded, subparallel to weakly sinuate in basal fifth, comparatively widely bordered with edges distinctly crenulate; hind angles right-angled to weakly obtuse, rounded at tips. Anterior half of disc with a vaguely defined longitudinal impression. Longitudinal lateral sulci distinctly convergent anteriorly, reaching anteriorly beyond half-length of pronotum; well defined transversal sulcus connecting deep pores; area between transverse sulcus and basal, shallow depression/sulcus weakly convex, with a guitar-shaped shallow, median depression; posterior margin distinctly lobed at mid-line. Prosternal process widely separates front coxae, widest at mid length (Fig. 6e).

Elytra 0.82–0.88 mm long, 1.38–1.45 times longer than wide; 2.60–2.70 times as long as and 1.25 times as wide as pronotum; widest at basal fourth then continuously distinctly converging to rounded apex; with hooked tooth present at anterolateral corner. Punctation composed of small setiferous punctures, and sparse larger, shallow foveate punctures (Fig. 6d). Hind wings well developed, 1.3 times longer than elytra.

Legs moderately long. Femora very narrow at base, strongly widened at apical half. Tibiae narrow, straight, continuously widened towards their apices. Metatibiae very narrow, 0.35 times as long as elytra. Metatarsus moderately long, 0.55 times as long as metatibia.

Abdomen with ventrite 1 slightly shorter than metaventrite and as long as three following ventrites combined (Fig. 6f). Ventrite 5 arcuate at apex.

Female genitalia with long, narrow coxites, emarginate at their apices, styli indistinct; spermatheca with one chamber rounded and second irregularly long oval; sperm duct moderately long, accessory gland elongate oval (Fig. 3d, e).

Male unknown.

##### Type material.

**Holotype**, **female**, **BOLIVIA**, “BOLIVIA: Cochabamba Dept. Est. Biol. Sacta, Univ Mayor S. Simeon. 17°06.48', 64°46.94', 300 m; rainforest FIT; 16/27-XII-2005; s. & J. Peck 05-47” (MNKM). **Paratypes**, **BOLIVIA**, same data as holotype (3 females: FSCA; 1: MIZ; 1: NMPC): **BRAZIL**, “Brazil: Manaus, AM. INPA/ Smithsonian Res. 2°25'S, 59°50'W, R. Didham. III.1994/ Leaf litter, Winkler method, Terra firmé fst./ BMNH(E), 2003-84/ 900. 2/ Main series in INPA, Manaus Brazil/ Lycoperdinella sp./ 0426 [pink label] (1 female: BMNH); “Brazil: Manaus, AM. INPA/ Smithsonian Res. 2°25'S, 59°50'W, R. Didham. III.1994/ Leaf litter, Winkler method, Terra firmé fst./ BMNH(E), 2003-84/ 320. 1 (1 female: BMNH); same but 770. 5 (1 female: MIZ).

##### Distribution.

South America: Bolivia (Cochabamba), Brazil (Manaus) (Fig. 21).

##### Comment.

Robertson et al. (2015) included in their molecular analysis one specimen from Bolivia, Santa Cruz province, identified as *Lycoperdinella subcaeca*. However, we have studied pictures of the disarticulated voucher and concluded that it certainly does not belong to that species. Most likely it is a member of *L. boliviensis* n. sp.

Fragments of phragmospores (Fig. 3d) found in the gut content of one Bolivian specimen of *L. boliviensis* suggest that this species probably feeds on Ascomycetes fungi.

### Key to species of *Lycoperdinella*

**Table:** 

1	Pronotum 0.80 times as long as wide (Fig. 5b); eye composed of six facets (Fig. 5a); antennal grooves on head absent; labium with anterior margin of mentum sharply produced medially (Fig. 5a); mesoventral process 1.1 times as wide as mesocoxal diameter (Fig. 5f); wingless; Guatemala, Costa Rica (?)	***L. subcaeca* Champion**
–	Pronotum 0.68–0.70 times as long as wide (Fig. 6c); eye composed of 36 facets (Fig. 6a, b); antennal grooves on head weakly marked; labium with anterior margin of mentum straight (Fig. 6b); mesoventral process 0.9 times as wide as mesocoxal diameter (Fig. 6e); hind wings well developed; Bolivia, Brazil	***L. boliviensis* sp. n.**

#### Rueckeria gen. n.

Taxon classificationAnimaliaColeopteraEndomychidae

http://zoobank.org/7EAC6A5C-C1FC-43D5-A3C9-CF6DD24C8D4D

Figs 7 , 8 , 9 , 10 , 11 , 12 , 13 , 14 , 15 , 16 , 17 , 18 , 19 , 20 , 21

##### Type species.

*Rueckeria inecol* sp. n.

##### Etymology.

This genus is dedicated to Dr. Wolfgang Rücker, German coleopterist, who has devoted many years of his life to the study of merophysiine beetles.

Gender feminine.

##### Diagnosis.

**Figure 7.**
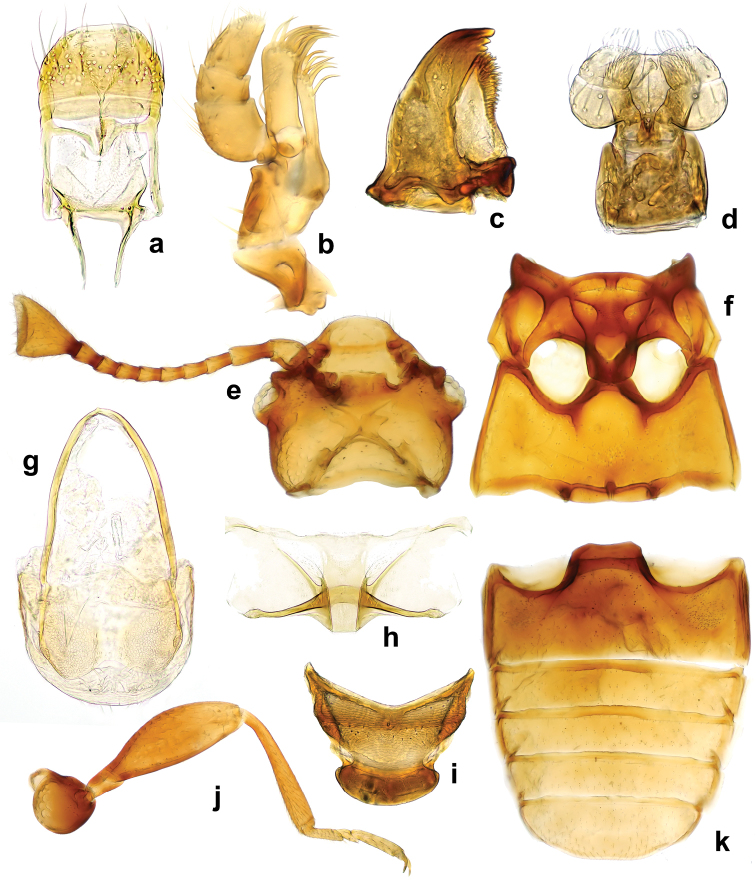
*Rueckeria* gen. n. and spp. n. **a–b**, **e–k** *Rueckeria inecol* sp. n. **c–d** *Rueckeria nigrileonis* sp. n. **a** labrum **b** maxilla **c** mandible **d** labium **e** ventral view of head with bucal parts removed **f** meso- and metaventrite **g** Male genital segment **h** metanotum **i** scutellum **j** mid leg **k** abdominal ventrites.

*Rueckeria* can be easily distinguished from other Neotropical Merophysiinae by the following combination of characters: antenna 10-segmented with 1-segmented club (Fig. 7e); antennal grooves on head absent or indistinct (Figs 16a, 17a, 18a, 19b, 20a); pronotum with lateral margins narrowly bordered and weakly crenulate (Figs 16c, 17b, 18e, 19b, d, 20b, c); metaventrite without postcoxal lines (Fig. 7f); abdominal ventrite 1 with arcuate, closed postcoxal lines (Figs 7k, 12g, 16f, 17f, 18g, 19g); hind wings absent. *Rueckeria* is most similar to *Lycoperdinella* and *Holoparamecus* but from *Lycoperdinella* it can be differentiated by the lateral margins of the pronotum at most weakly crenulate (coarsely crenulate in *Lycoperdinella*), elytra with anterolateral corners simple (Figs 16d, 17d) (with hooked tooth on each elytron in *Lycoperdinella*), postcoxal lines present on abdominal ventrite 1 (postcoxal lines absent in *Lycoperdinella*), longer and more slender antennae, the body covered with much shorter setae (long setae in *Lycoperdinella*) and the hind wings always absent. From *Holoparamecus*, *Rueckeria* can be separated by having lateral margins of the pronotum bordered (smooth in *Holoparamecus*), pronotum narrower near base but not distinctly constricted basally as in *Holoparamecus*, antenna with 1-segmented club (2-segmented club present in *Holoparamecus*) and postcoxal lines present on abdominal ventrite 1 (postcoxal lines absent in *Holoparamecus*).

##### Description.

Length 1.3–2.2 mm. Body elongate, approx. 2.0 times longer than wide, weakly convex, 2.8–3.3 times as long as high; shiny, smooth, covered with sparse and short pale setae. Color light brown to black.

Head (Fig. 7e) deeply retracted in prothorax (Fig. 15b), slightly wider than long; sparsely and moderately densely punctate. Gular sutures subparallel, widely separated. Eyes very small, oval, coarsely faceted, composed of 16–18 facets (Fig. 18a). Antennal sockets concealed by sides of frons, not visible from above; antennal grooves absent. Antenna moderately long (Figs 7e, 18c, 19c), surpassing base of pronotum, composed of 10 antennomeres with club formed by terminal antennomere which is large, inflated, subtriagular, obliquely truncate at apex. Frontoclypeal suture weakly arcuate (Fig. 7e). Clypeus transverse, flat, convergent anteriorly. Labrum (Fig. 7a) subquadrate, with rounded anterolateral corners, truncate apically, with submembranous apex, punctate, covered with sparse, long setae; tormae with mesal arms recurved posteriorly; labral rods absent. Mandible (Fig. 7c) with two apical teeth and with four smaller subapical teeth getting subsequently smaller posteriorly; prostheca covered with digitiform setae on anterior third getting thiner and shorter toward posterior 2/3; mola large, sclerotized; submola small, membranous. Maxilla (Fig. 7b) with palpomere 1 and 3 very short; palpomere 2 large and swollen, approx. twice as long as palpomere 3; terminal palpomere 1.5 times as long as 2 and 3 palpomeres combined, narrow, tapering, apex obliquely truncate to weakly rounded; galea moderately broad, twice as broad as lacinia, with long, broad, apical spine-like setae; lacina elongate, with spine-like setae on apical half. Labium (Fig. 7d) with palpomere 1 very small; palpomere 2 largest, oval, inflated; terminal palpomere subquadrate, truncate at apex. Mentum subquadrate (Figs 16b, 17a, 18a, 19b, 20a), with produced anterior angles; finely punctate, glabrous. Prementum subquadrate, sclerotized, with apically expanded, membranous ligula. Tentorium (Fig. 7e) with anterior arms fused medially and widely divergent anteriorly; corpotentorium absent.

Prothorax. Pronotum (Figs 16c, 17b, 18b, 19e, 20b) weakly transverse, widest at anterior 1/4; pronotal surface finely and sparsely punctate; lateral edges narrowly bordered, weakly crenulate, arcuately widening at anterior 1/4, and almost parallel at basal 1/4; anterior margin curved with slightly projected rounded angles; posterior angles almost right-angled. Pronotal disc weakly convex. Pronotal base with an impression composed of two longitudinal sharply defined, slightly convergent sulci reaching almost anterior third, and a pair of deep transverse depressions/sulci. Anterior transverse sulcus flanked by one small deep fovea on each side, fovea sometimes absent (Fig. 20c); area between transverse sulci convex, basal sulcus not reaching lateral sulci, provided with large, shallow foveate punctures. Prosternal process (Figs 16e, 17e) broad, approx. as wide as coxal diameter, with raised margins; extending posteriorly beyond front coxae. Procoxa circular in outline its cavity externally open behind, internally closed; trochantin concealed.

Meso- and metathorax. Mesonotum (Fig. 7h) sclerotized; scutellar shield small, strongly transverse, widely rounded apically, partially covered by base of pronotum. Mesoventrite (Figs 7f, 16e, 17e) carinate at middle; intercoxal process moderately elongate, rather broadly separates mesocoxae (slightly narrower than coxal diameter) reaching half of its length. Mesoventrite fused with mesanepisternum (trace of suture visible). Mesocoxe weakly oval in outline, its cavity narrowly closed outwardly by sterna; trochantin concealed. Meso-metaventral junction of straight-line type, without internal knobs. Elytron elongate, convex, irregularly and moderately finely punctate (Fig. 18e), border at anterolateral corner without teeth (Figs 16d, 17d); epipleuron narrow, incomplete at apex. Sutural stria sharply defined (Fig. 18e), complete, widest at mid length, then weakly converging towards elytral apex. Metaventrite (Figs 7f, 16e, 18f, 20d) transverse, twice as long as mesoventrite, weakly convex; anterior margin thick, with postcoxal longitudinal ridges reaching between anterior 1/3 to 2/5; discrimen very short. Metacoxae transversely oval, widely separated. Metendosternite with very short stalk and moderately widely separated anterior tendons. Hind wing absent.

Legs. Trochanter moderately elongate (Fig. 7j); trochanterofemoral attachment oblique. Femur widest near middle of its length, more than twice as wide as tibia, sparsely setose; tibia and tarsus covered with long, dense setae. Tibia narrow, straight or slightly bent inwards, continuously weakly widened distally or with abrupt wider part at distal third, without apical spurs. Tarsal formula 3-3-3 in both sexes (Fig. 17d): tarsomere 1, 1.5 times longer than 2; tarsomere 3 slightly longer than remaining tarsomeres combined. Claws simple. Empodium very small.

Abdomen (Figs 7k, 12g, 16f, 17f, 18g, 19g) with five freely articulated ventrites; ventrite 1 slightly longer than three following ventrites together, with arcuate, complete postcoxal lines; ventrites 2–4 almost equal in length; ventrite 5 long, acuminate, approx. as long or shorter than ventrites 3–4 together.

**Figure 8.**
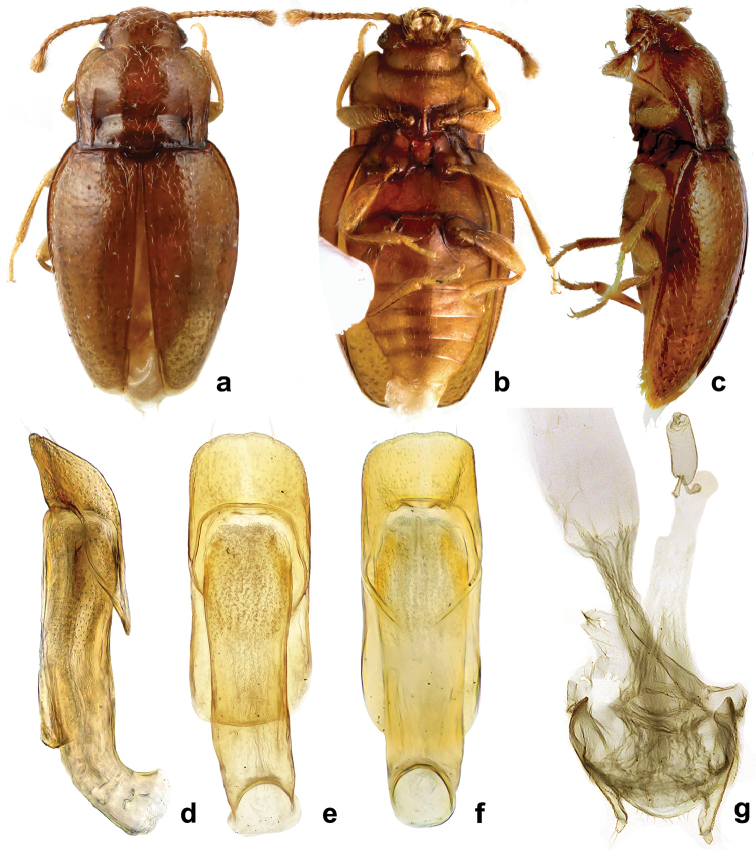
*Rueckeria inecol* sp. n. **a** dorsal habitus **b** ventral habitus **c** lateral habitus **d** lateral view of aedeagus **e** dorsal view of aedeagus **f** ventral view of aedeagus **g** female genitalia.

**Figure 9.**
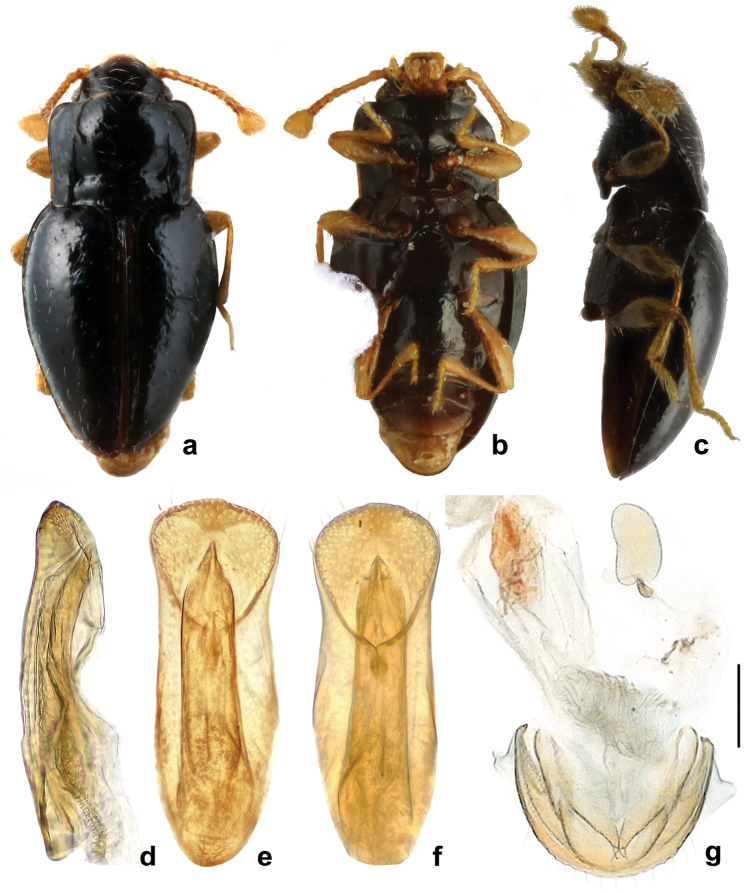
*Rueckeria nigrileonis* sp. n. **a** dorsal habitus **b** ventral habitus **c** lateral habitus **d** lateral view of aedeagus **e** dorsal view of aedeagus **f** ventral view of aedeagus **g** female genitalia.

**Figure 10.**
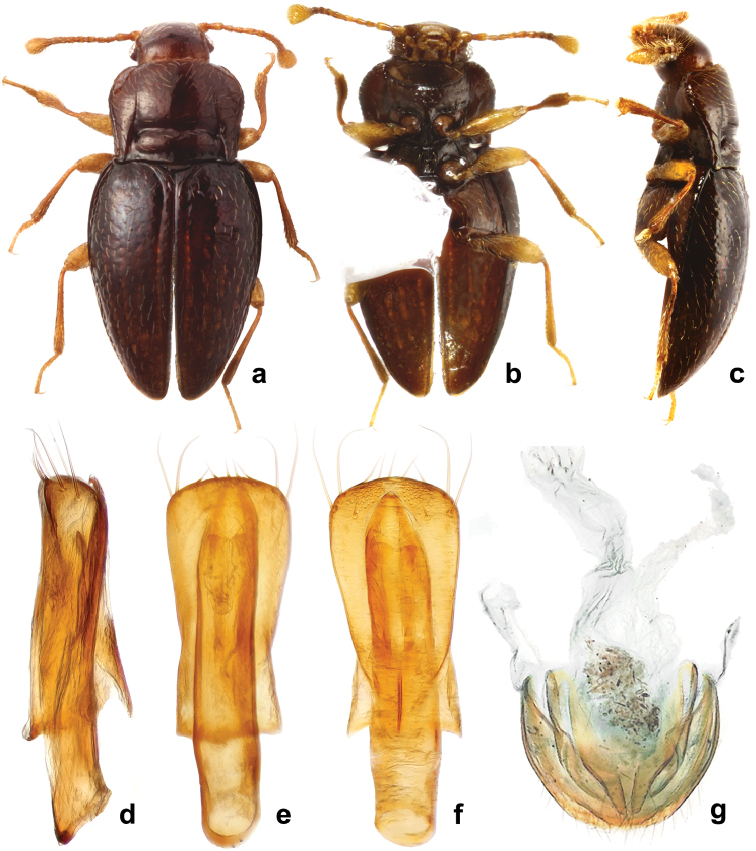
*Rueckeria ocelotl* sp. n. **a** dorsal habitus **b** ventral habitus **c** lateral habitus **d** lateral view of aedeagus **e** dorsal view of aedeagus **f** ventral view of aedeagus **g** female genitalia.

**Figure 11.**
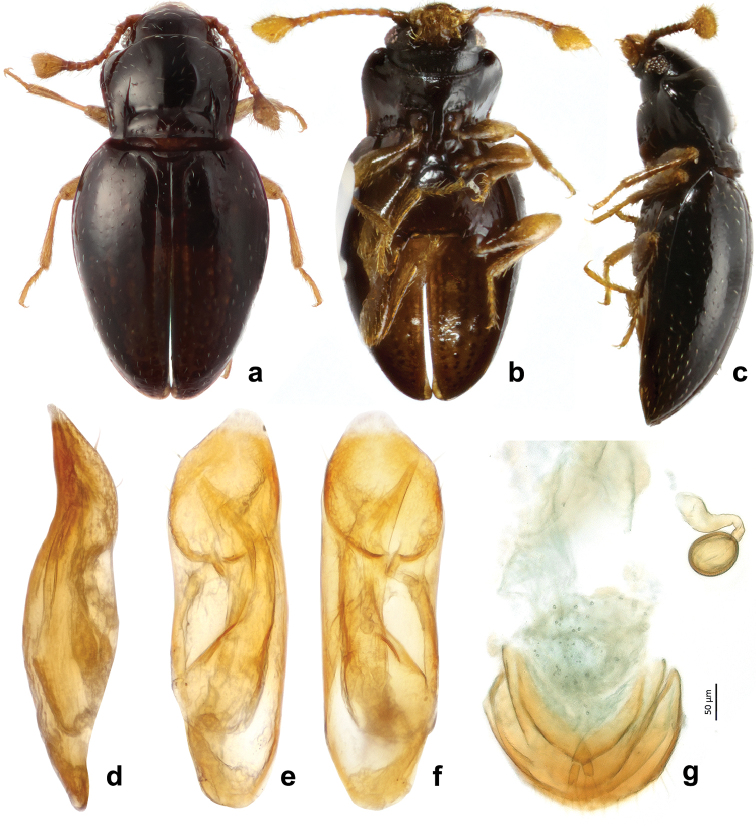
*Rueckeria puma* sp. n. **a** dorsal habitus **b** ventral habitus **c** lateral habitus **d** lateral view of aedeagus **e** dorsal view of aedeagus **f** ventral view of aedeagus **g** female genitalia.

**Figure 12.**
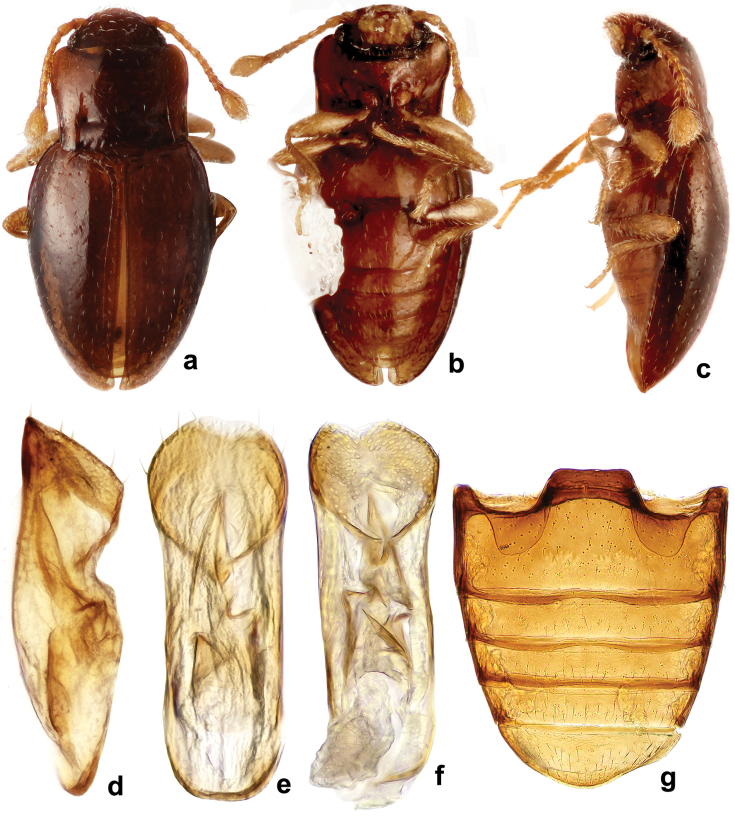
*Rueckeria skelleyi* sp. n. **a** dorsal habitus **b** ventral habitus **c** lateral habitus **d** lateral view of aedeagus **e** dorsal view of aedeagus **f** ventral view of aedeagus **g** abdominal ventrites.

Aedeagus (Figs 8d–f, 9d–f, 10d–f, 11d–f, 12d–f) resting on its side when retracted. Median lobe stout, with basal 2/3 strongly to weakly curved. Tegmen large, slightly shorter to 1.5 times longer than median lobe; parameres fused; tegminal strut absent or present.

Female genitalia (Figs 8g, 9g, 10g, 11g). Ovipositor weakly sclerotized, with coxites elongate; styli well developed or vestigial, placed apically. Spermatheca moderately large, elongate oval, submembranous; sperm duct moderately long, slender, membranous with short part connected to spermatheca sclerotized; accessory gland small membranous, elongate.

##### Distribution.

Mexico: Hidalgo, Querétaro, Veracruz (Fig. 21).

#### Rueckeria inecol sp. n.

Taxon classificationAnimaliaColeopteraEndomychidae

http://zoobank.org/2117C9E1-B350-4299-81DB-19C098A9D9AB

**Figure 13.**
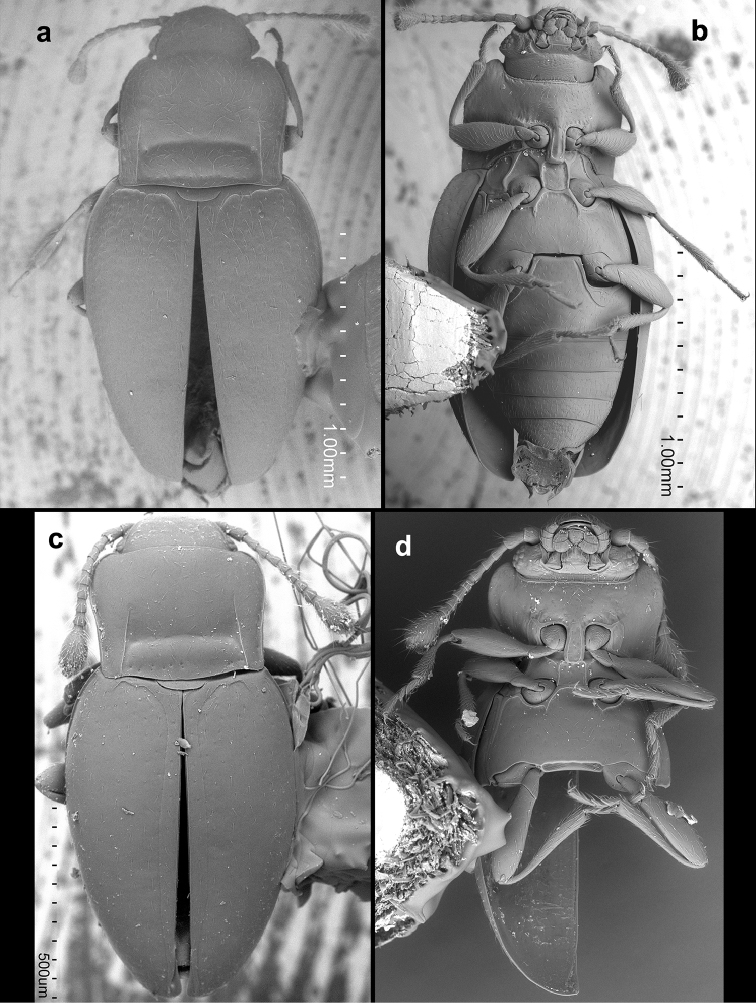
*Rueckeria* species **a–b** *Rueckeria inecol* sp. n. **c–d** *Rueckeria skelleyi* sp. n. **a** dorsal view **b** ventral view **c** dorsal view **d** ventral view.

Figs 7a–b, e–k , 8 , 13a–b , 16

##### Etymology.

The name of the new species is dedicated to our colleagues in INECOL (The Institute of Ecology, Xalapa, Mexico), institution where the project within most specimens of this species were collected was held. Noun in apposition.

##### Differential diagnosis.

*Rueckeria inecol* is similar to *R. skelleyi* and *R. ocelotl* spp. n., by having the body completely brown and the abdominal ventrite 1 with irregularly rounded postcoxal lines (Fig. 16f). However, it can be distinguished by the basal pronotal pores present (Fig. 16c) (not perforated in *R. skelleyi*), mentum subquadrate (Fig. 16b) and pronotal lateral margins nearly smooth (Fig. 16c) (mentum subhexagonal and pronotal margins weakly crenulate in *R. ocelotl*), and by the features of the aedeagus (Fig. 8d–f), with the tegmen about as long as the median lobe, with the sides parallel, and the median lobe widening apically.

##### Description.

Length 1.90–2.17 mm, width 1.00–1.05 mm, height 0.62–0.67 mm; body elongate-oval, weakly convex, 2.07–2.34 times as long as wide, 3.32–3.36 time as long as high (Figs 8a–c, 13a, b). Surfaces shiny; sparsely covered with short, decumbent golden setae. Color reddish brown with yellowish brown antennae and legs. Head with interocular distance 0.77 times as wide as head including eyes (Fig. 16a, b). Eyes small, composed of 16 facets. Antenna moderately long and slender (Fig. 7e), 0.86 times as long as head and pronotum combined; scape as long as wide, 1.7 times as long as pedicel; pedicel 1.8 times longer than wide; antennomeres 3–5, each 1.42 times as long as wide, 0.7 times as long as pedicel; antennomeres 6–8, 1.2 times as long as wide and 0.5 times as long as pedicel; antennomere 9, 1.33 times as long as wide, 0.55 times as long as pedicel; terminal antennomere inflated, asymmetrical, 2.2 times longer at longer margin than pedicel, longer margin 1.28 times as long as lateral one, apical margin concave. Mentum subquadrate, with straight anterior margin (Fig. 16b).

Pronotum weakly transverse (Fig. 16c), 0.75–0.80 times as long as wide, 1.55 times as wide as head, 1.05–1.06 times wider at widest part than at base, widest at anterior fourth, convex at mid length; front angles rounded, weakly produced, margins slightly sinuate, narrowing at basal third; lateral margins narrowly bordered, edges nearly smooth; hind angles right-angled to weakly obtuse, rounded at tips. Anterior half of disc without impressions. Longitudinal, lateral sulci weakly convergent, almost reaching anterior 2/5; basal lateral pores present, connected by deep, faintly defined transversal sulcus, posterior transverse sulcus (near posterior margin) provided with foveate punctures; area between transverse sulci convex. Prosternal process moderately widely separates front coxae (Fig. 16e), weakly widening posteriad, its apical width 0.85 times length of procoxae.

Elytra 1.25–1.32 mm long, 1.24–1.28 times as long as wide; 2.37–2.50 times as long and 1.44–1.53 times as wide as pronotum; widest at basal fourth, then continuously strongly converging to rounded apex. Punctation composed of widely spaced small setiferous punctures and slightly larger, shallow foveate punctures (Fig. 16d). Metaventrite with postcoxal longitudinal ridges reaching shortly before anterior 2/5 (Fig. 16e).

Legs moderately long. Femora very narrow at base, then strongly widened at apical half. Tibiae moderately narrow, continuously widening to apex. Metatibia very narrow, continuously widening apically, 0.31–0.36 times as long as elytra; metatarsus long, 0.66 times as long as metatibia.

Abdomen with ventrite 1 slightly shorter than metaventrite and almost as long as three following ventrites combined (Figs 7k, 16f); postcoxal lines on ventrite 1 irregularly rounded, reaching about 1/3 length of ventrite.

Male genital segment with sternite emarginate apically and acuminately rounded at its base (Fig. 7g). Tegmen large, parallel sided, approx. as long as median lobe; tegminal strut short. Median lobe cylindrical, distinctly curved at base, weakly narrowing apically, with apex gently rounded (Fig. 8d–f).

Female genitalia with narrow coxites, with moderately large styli bearing two apical setae; spermatheca elongate (Fig. 8g).

##### Type material.

**Holotype male**, **MEXICO**, “Mexico: Veracruz Trucha Feliz, 1 km SW of Rancho Viejo (W of Xalapa) 19°31.1'N 96°59.1'W; 1445 m 9.ix.2016; Alvarado, Arriaga, Fikáček & Seidel lgt. 2016-MX15 / sifting of accummulations of leaf litter in small pieces of riverside forest” (NMPC).)**Paratypes**, “MEXICO: Veracruz, Coatepec, Reserva La Cortadura, 12 km NW Coatepec town. 1900-2000 m fragmented cloud forest. Sifted leaf litter, Berlese funnel. 20.VI.2012. F. Alvarado & R. Madrigal” (1 male: IEXA; 1 male: CNIN; 1 female: IEXA); “Mexico: Veracruz. Tlalnehuayocan, Rio Pixquiac, Fragmented cloud forest, 1522 m, 19°32'49"N 96°59'52"W, 5.V.2013, Leg. E. Arriaga, F. Alvarado & R. Madrigal.” (1 female: MIZ).

##### Distribution.

Mexico: Veracruz (Fig. 21).

#### Rueckeria puma sp. n.

Taxon classificationAnimaliaColeopteraEndomychidae

http://zoobank.org/B46FEB44-015C-4366-8B49-175CC6E74E99

**Figure 14.**
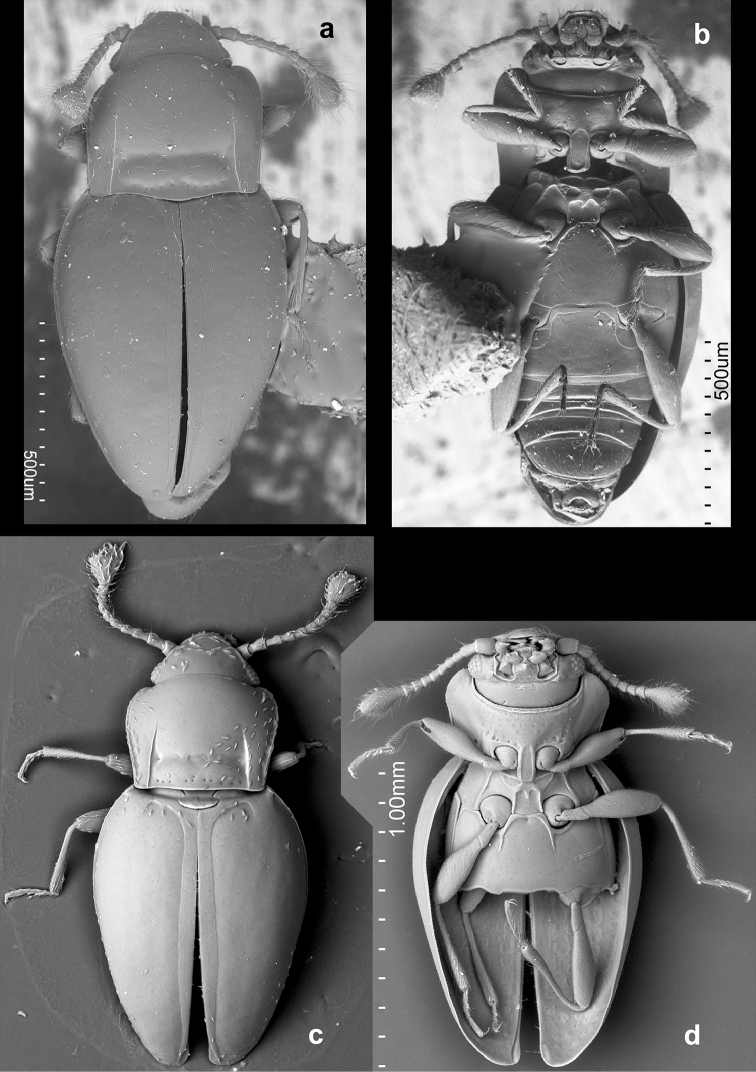
*Rueckeria* species **a–b** *Rueckeria nigrileonis* sp. n. **c–d** *Rueckeria puma* sp. n. **a** dorsal view **b** ventral view **c** dorsal view **d** ventral view.

Figs 11a–g , 14c, d , 19

##### DNA barcode.

GenBank accession number: MG676234

##### Etymology.

The name of this new species is dedicated to our coleopterist colleagues in the National Collection of Insects in UNAM (National Autonomous University of Mexico) whose mascot is the puma, the pan-American felid.

##### Differential diagnosis.

*Rueckeria puma* is most similar to *R. nigrileonis* by its small size, black body with yellow legs, antennae, and mouth parts (Fig. 11a–c); however, it can be distinguished by the pronotum more narrowed at base (1.20 times as wide at widest part than at base), abdominal postcoxal lines irregularly rounded and deeper reaching about half length of the ventrite 1 (Fig. 18g), and the aedeagus with the tegmen longer than the median lobe with the apex acuminate and the median lobe strongly widened at base and curved (Fig. 11d–f).

##### Description.

Length 1.15–1.60 mm, width 0.72–0.54 mm, height 0.41 mm; body moderately elongate-oval, moderately convex, 2.2–2.1 times as long as wide, 3.1–3.0 times as long as high (Figs 11a–c, 14c, d) . Surfaces shiny, sparsely covered with very short, decumbent, golden setae. Color mainly black with ventral surfaces infuscate yellow, clypeus reddish brown, and mouth-parts and legs yellowish.

Head with interocular distance 0.8 times as wide as head including eyes (Fig. 19a). Eyes small, composed of 18 facets. Antenna moderately long and slender (Fig. 19c), 0.85 times as long as head and pronotum combined; scape 1.30 times longer than wide, as long as pedicel; pedicel 1.55 times longer than wide; third antennomere 1.58 times longer than wide, subequal in length with pedicel; antennomeres 4–8 getting gradually shorter and wider towards antennomere 9 which is as long as wide and 0.5 times longer than pedicel; terminal antennomere inflated, asymmetrical, 2.5 times as long at longer margin as pedicel, its longer margin 1.22 times longer than lateral one and 1.4 times longer than apical margin, apical margin truncate. Mentum rectangular (Fig. 19b), weakly produced anteriorly in middle of apical margin.

Pronotum weakly transverse (Fig. 19e), 0.78 times as long as wide, 1.38 times wider than head, approx. 1.2 times wider at widest part than at base, widest at anterior fourth, strongly convex in mid length; front angles rounded, weakly produced, margins slightly sinuate, narrowing at basal third, narrowly bordered with edges very weakly crenulate; hind angles right-angled to weakly obtuse, rounded at tips. Anterior half of disc without impressions. Longitudinal sulci weakly convergent, reaching nearly anterior 2/5 of pronotum; basal lateral pores present, connected by deep, faintly defined transversal sulcus; posterior transverse sulcus provided with large foveate punctures, area between transverse sulci convex, weakly depressed at mid-line; posterior margin weakly lobed at mid-line. Prosternal process widely separates front coxae (Fig. 19f), widest at mid length.

Elytra 0.88–0.97 mm long, 1.30–1.38 times longer than wide; 2.30–2.38 times as long as and 1.38–1.42 times as wide as pronotum; widest at basal fourth then continuously strongly converging to rounded apex. Punctation composed of small setiferous punctures, each accompanied posteriorly by 2–3 slightly larger shallow foveate punctures. Metaventrite with postcoxal longitudinal ridges extending slightly beyond anterior 2/5 (Fig. 19f).

Legs moderately long. Femora very narrow at base, strongly widened at apical half. Pro- and mesotibiae very narrow, protibiae weakly sinuate, slightly widened towards apex. Metatibia very narrow, almost straight, continuously widening apically, more accentuated at apical fifth, slightly bent inwards in apical 2/3, 0.39–0.40 times as long as elytra. Metatarsus long, 0.65 times as long as metatibia.

Abdomen with ventrite 1 slightly shorter than metaventrite and as long as three following ventrites combined (Fig. 19g); postcoxal lines on ventrite 1 reaching about half length of ventrite, weakly irregularly rounded. Ventrite 5 rounded apically.

Male genital segment with sternite emarginate apically, and acuminately rounded at its base. Tegmen large, sinuate in lateral view, with rugose and distinctly acuminate apex. Median lobe short, strongly widened at base, markedly curved, continuously strongly narrowing to acute apex. Tegminal strut absent (Fig. 11d–f).

Female genitalia (Fig. 11g) with moderately broad coxites, and with large, elongate styli bearing two apical setae; spermatheca oval.

##### Type material.

**Holotype, male, MEXICO**, “MEXICO: Hidalgo La Mojonera 4.8 km SE Zacualtipan, upper part of *Fagus* forest; 20°37.9'N 98°37.0'W; 2010 m; 13-16.ix.2016; Arriaga, Cortés, Fikáček & Seidel lgt. 2016-MX22 / sifting of large accummulations of leaf litter in relictual Fagus forest with intermixed Magnolia and tree ferns, with sparse to dense understory and many (partly rotten) fungi and logs” (CNIN). **Paratype**. “MEXICO: Hidalgo La Mojonera 4.4 km SE Zacualtipan, lower part of *Fagus* forest; 20°38.0'N 98°37.1'W; 1940 m; 14-16.ix.2016; Cortés, Fikáček & Seidel lgt. 2016-MX24 / sifting of large accumulations of leaf litter in *Fagus* forest above the small stream” (1 female: NMPC).

##### Distribution.

Mexico: Hidalgo (Fig. 21)

#### Rueckeria nigrileonis sp. n.

Taxon classificationAnimaliaColeopteraEndomychidae

http://zoobank.org/CDBDDEB8-0A3E-4629-84A2-5C2452C68C6D

Figs 7c, d , 9 , 14a, b , 17

##### DNA barcode.

GenBank accession number: MG676232

##### Etymology.

This new species is dedicated to our coleopterist colleagues in the entomological collection of the University of Guadalajara, whose mascot is a black lion. The name is derived from Latin “*niger*” (black) and “*leo*” (lion).

##### Differential diagnosis.

*Rueckeria nigrileonis* is most similar to *R. puma* by its small size and black body with legs, antennae and mouth parts yellow (Fig. 9a–c). However it can be distinguished by the pronotum being less narrowed at base (1.05 times wider at widest part than at base) (Fig. 17b), abdominal postcoxal lines regularly rounded, shallower, reaching about 1/3 length of ventrite 1, and the aedeagus with the tegmen longer than the median lobe with apex rounded and the median lobe moderately widened at base and curved (Fig. 9d–f).

##### Description.

Length 1.29–1.37 mm, width 0.67–0.71 mm, height 0.5 mm; body moderately elongate-oval, moderately convex, 1.92–1.95 times as long as wide, 2.80 –2.95 times as long as high (Figs 9a–c, 14a, b). Surfaces shiny, sparsely covered with very short, decumbent, golden setae. Color mainly black with ventral surfaces infuscate yellow, clypeus reddish brown and mouth parts and legs yellowish.

Head with interocular distance 0.8 times as wide as head including eyes. Eyes small, composed of 18 facets. Antenna moderately long and slender, 0.85 times as long as head and pronotum combined; scape 1.33 times longer than wide, 1.33 times as long as pedicel; pedicel 1.36 times longer than wide; third antennomere 1.66 times longer than wide, equal in length with pedicel; antennomeres 4–8 getting gradually shorter and wider towards antennomere 9 which is as long as wide and 0.7 times longer than pedicel; terminal antennomere inflated, asymmetrical, 2.25 times as long at longer margin as pedicel, its longer margin 1.45 times longer than lateral one and 1.05 as long as apical margin, apical margin truncate. Mentum subquadrate, weakly produced anteriorly in middle of anterior margin (Fig. 17a).

Pronotum weakly transverse (Fig. 17b), 0.75–0.80 times as long as wide, 1.41 times wider than head, 1.05 times wider at widest part than at base, widest at anterior fourth, strongly convex in mid length; front angles rounded, weakly produced; lateral margins slightly sinuate, narrowing at basal third, narrowly bordered with edges very weakly crenulate; hind angles right-angled to weakly obtuse, rounded at tips. Anterior half of disc without impressions. Longitudinal sulci weakly convergent, reaching nearly half length of pronotum; basal lateral pores present, connected by deep, faintly defined transversal sulcus; posterior transverse sulcus provided with large foveate punctures, area between transverse sulci convex, weakly depressed at mid-line; posterior margin weakly lobed at mid-line. Prosternal process widely separates front coxae, widest at mid length (Fig. 17e).

Elytra 0.80–0.88 mm long, 1.18–1.24 times longer than wide; 2.10–2.20 times as long and 1.31–1.38 times as wide as pronotum; widest at basal fourth then continuously strongly converging to rounded apex. Punctation composed of small setiferous punctures, each accompanied posteriorly by 2–3 slightly larger shallow foveate punctures (Fig. 17c).

Legs moderately long. Femora very narrow at base, strongly widened at apical half. Pro- and mesotibiae very narrow, slightly curved inwards, parallel sided along basal 2/3, then widened towards apex. Metatibia very narrow, almost straight, continuously widening apically, more accentuated at apical fifth, 0.38–0.42 times as long as elytra. Metatarsus long, 0.68 times as long as metatibia.

Abdomen with ventrite 1 slightly shorter than metaventrite and as long as three following ventrites combined (Fig. 17f); postcoxal lines on ventrite 1 weakly asymmetrically rounded, complete. Ventrite 5 rounded at apex.

Male genital segment with sternite emarginate apically, and acuminately rounded at its base. Tegmen large, slightly curved in lateral view, with rugouse apex. Median lobe short, markedly curved, continuously strongly narrowing to acute apex. Tegminal strut absent (Fig. 9d–f).

Female genitalia with moderately broad coxites with two apical setae, styli vestigial; spermatheca large, subreniform (Fig. 9g).

##### Type material.

**Holotype, male, MEXICO** “Mexico: Veracruz. Tlalnehuayocan, Río Pixquiac, Fragmented cloud forest, 1522 m 19°32'4.9"N 96°59'52"W, Sifted leaf litter, 5.V.2013. Leg. E. Arriaga, F. Alvarado & R. Madrigal (NMPC). **Paratypes**, “Mexico: Veracruz Trucha Feliz, 1 km SW of Rancho Viejo (W of Xalapa) 19°31.1'N 96°59.1'W; 1445 m 9.ix.2016; Alvarado, Arriaga, Fikáček & Seidel lgt. 2016-MX15 / sifting of accumulations of leaf litter in small pieces of riverside forest” (1 male: CZUG); “Mexico: Veracruz, Coatepec, Reserva La Cortadura, 12 km NV Coatepec town, 1900-2000 m, Fragmented cloud forest, Sifted leaf litter, Winkler extractor, 20.VI. 2012, F. Alvarado & R. Madrigal” (1 female: MIZ); “MEXICO: Veracruz, San Andrés Tlalnehuayocan, 19°31'00.0"N, 97°00'25.4"W. 1500 m. Fragmented cloud forest. Sifted leaf litter, Winkler extractor. 13.VI.2012. Leg. F. Alvarado & R. Madrigal” (1 male: IEXA).

##### Distribution.

Mexico: Veracruz (Fig. 21).

#### Rueckeria skelleyi sp. n.

Taxon classificationAnimaliaColeopteraEndomychidae

http://zoobank.org/DB845616-B120-457B-842D-5A25C9A759CB

Figs 12 , 13c, d , 20

##### Etymology.

The name of the new species is dedicated to our colleague Dr. Paul Skelley, the curator of the entomology collection in FSCA, where the holotype of this species was found during a visit of EA-V and WT in this collection.

##### Differential diagnosis.

*Rueckeria skelleyi* is similar to *R. inecol* and *R. ocelotl* spp. n, by the body completely brown and the abdominal ventrite 1 with irregularly rounded postcoxal lines. However it can be distinguished by the basal lateral pores not perforated as in the other species (Fig. 20c), mentum subrectangular and pronotal lateral margins smooth (Fig. 20c) (mentum subhexagonal and pronotal margins weakly crenulate in *R. ocelotl*), and by the features of the aedeagus (Fig. 12d–f): tegmen markedly longer than the median lobe which is widened at base and provided with an additional acute process ventrally.

##### Description.

Length 1.50 mm, width 0.64 mm, height 0.43 mm; body elongate-oval, weakly convex, 2.35 times as long as wide, 3.32–3.50 times as long as high (Figs 12a–c, 13c, d). Surfaces shiny, sparsely covered by short, decumbent golden setae. Color reddish brown with yellowish brown antennae and legs.

Head with interocular distance 0.80 times as wide as the head including eyes. Eyes small, composed of approximately 16 facets. Antenna moderately long and slender, 0.75 times as long as the head and pronotum combined; scape 1.20 times as long as wide, 1.16 times as long as pedicel; pedicel 1.5 times longer than wide; third antennomere 1.8 times as long as wide, 0.83 times as long as pedicel; antennomeres 4–7, 1.4 times as long as wide and 0.5 times as long as pedicel; antennomeres 8–9 as long as wide, 0.7 times as long as pedicel; terminal antennomere inflated, asymmetrical, 3.0 times longer at longer margin than pedicel, longer margin 1.32 times longer than lateral one, apical margin truncate. Mentum subquadrate, very weakly produced anteriorly in middle of anterior margin (Fig. 20a).

Pronotum weakly transverse (Fig. 20a), 0.76 times as long as wide, 1.4 times as wide as head, 1.07 times wider at widest part than at base, widest at anterior fourth, rather convex at mid length; front angles rounded, weakly produced, margins slightly sinuate, narrowing at basal third; margins narrowly bordered, weakly crenulate; hind angles right-angled (Fig. 20c), rounded at tips. Anterior half of disc without impressions. Longitudinal sulci weakly convergent, almost reaching apical 2/5; basal lateral pores not perforated (Fig. 20c), just present as shallow depressions conected by moderately deep, faintly defined transversal sulcus, posterior transverse sulcus shallow provided with large foveate punctures; area between transverse sulci convex. Prosternal process moderately widely separates front coxae, weakly widening posteriad, its apical width 0.75 times the length of procoxae (Fig. 20d).

Elytra 0.85 mm long, 1.40 times as long as wide; 2.43 times as long and 1.34 times as wide as pronotum; widest at basal fourth then continuously strongly converging to rounded apex. Punctation composed of small setiferous punctures and dispersed, slightly larger, shallow foveate punctures.

Legs moderately long. Femora very narrow at base, then strongly widened at apical half. Tibiae moderately narrow, continuously widening to apex. Metatibia very narrow, straight, continuously widening apically, 0.31–0.36 times as long as elytra; metatarsus very long, 0.66 times as long as metatibia.

Abdomen with ventrite 1 slightly shorter than metaventrite and almost as long as three following ventrites combined; postcoxal lines on ventrite 1 reaching about half length of ventrite, irregularly rounded. Ventrite 5 arcuate apically.

Male genital segment with sternite rounded apically, basal margin acuminately rounded. Tegmen large, distinctly longer than median lobe, parallel sided, weakly curved in lateral view, apex rounded; tegminal strut indistinct. Median lobe widened and with additional acute ventral process near base, then strongly narrowing to markedly acute apex (Fig. 12d–f).

Female unknown.

##### Type material.

**Holotype**, **male, MEXICO**, “MEXICO: Querétaro; Mpio: San Joaquin, Campo Alegre, 20°54'47"N, 99°34'35"W, 8-15-DEC-2013, 2480 m, P. Skelley, P. Kovarik, R. Jones, surface dung pitfall” (FSCA).

##### Distribution.

Mexico: Querétaro (Fig. 21).

#### Rueckeria ocelotl sp. n.

Taxon classificationAnimaliaColeopteraEndomychidae

http://zoobank.org/28805F2C-368D-497D-832C-E37835714DDE

**Figure 15.**
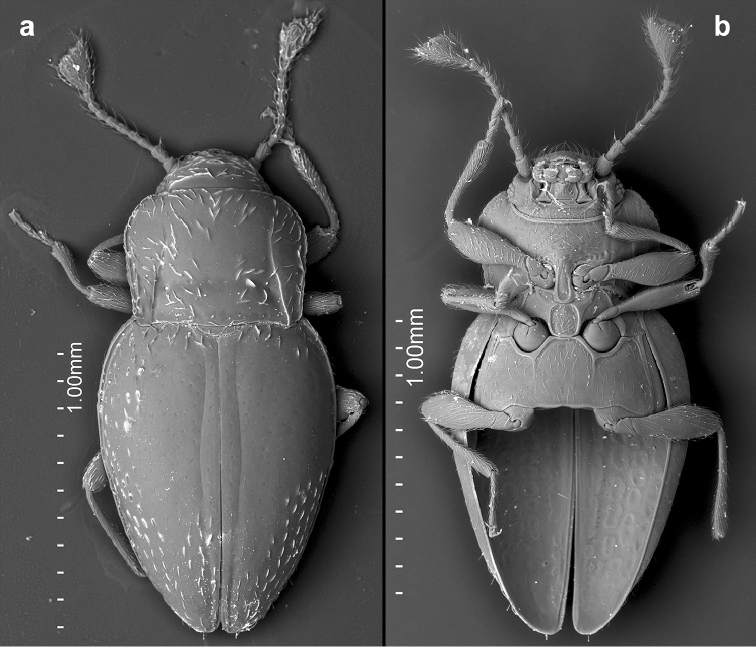
*Rueckeria ocelotl* sp. n. **a** dorsal view **b** ventral view.

Figs 10 , 15a, b , 18

##### DNA barcode.

GenBank accession number: MG676233

##### Etymology.

The name is derived from the Nahuatl word for jaguar, inspired by the rosette-like pattern that can be seen on the elytra under certain type of light, caused probably by different densities of the chitin. A noun in apposition.

##### Differential diagnosis.

*Rueckeria ocelotl* is similar to *R. inecol* and *R. skelleyi* spp. n, by the body completely brown and the abdominal ventrite 1 with irregularly rounded postcoxal lines (Fig. 18g). However, it can be distinguished by the pronotum with basal lateral pores present (not perforated in *R. skelleyi*), mentum subhexagonal (Fig. 18a) and pronotal lateral margins weakly crenulate (mentum subrectangular and pronotal margins smooth in *R. inecol*) and by the features of the aedeagus: tegmen distinctly shorter than median lobe, widened apically, median lobe subparallel-sided with moderately acuminate apex (Fig. 10d–f).

##### Description.

Length 2.00–2.15 mm, width 0.80–0.85 mm, height 0.62–0.67 mm; body elongate-oval, weakly convex, 2.35–2.50 times as long as wide, 3.32–3.36 time as long as high (Figs 10a–c, 15a, b). Surfaces shiny; sparsely covered with short, decumbent golden setae. Color reddish-brown with yellowish brown antenna and legs.

Head with interocular distance 0.75 times as wide as head including eyes. Eyes small, composed of approximately 16 facets (Fig. 18a). Antenna moderately long and slender (Fig. 18c), 0.78 times as long as head and pronotum combined; scape as long as wide, 0.8 times as long as pedicel; pedicel 2.0 times longer than wide; third antennomere 2.3 times as long as wide, 0.7 times as long as pedicel, antennomeres 4–5, 1.8 times as long as wide, 0.6 times as long as pedicel; antennomeres 6–8, 1.5 times as long as wide and 0.5 times as long as the pedicel; ninth antennomere 1.6 times as long as wide, 0.5 times as long as pedicel; terminal antennomere inflated, asymmetrical, 2.4 times longer at longer margin than pedicel, longer margin 1.4 times longer than lateral one, apical margin concave.

Pronotum weakly transverse, 0.77–0.80 times as long as wide (Fig. 18b), 1.51 times as wide as head, 1.09–1.14 times wider at widest part than at base, widest at anterior fourth, rather convex at mid length; front angles rounded, not conspicuously produced, margins slightly sinuate at anterior half, continuously narrowing to base; narrowly bordered, edges weakly crenulate (Fig. 18d); hind angles right-angled, rounded at tips. Anterior half of disc without impressions. Longitudinal sulci convergent, reaching apical 2/5; basal lateral pores present, connected by deep, faintly defined transversal sulcus, with additional posterior, transverse sulcus provided with large foveate punctures; area between transverse sulci convex. Prosternal process moderately widely separates front coxae, weakly widening posteriad, its apical width 0.85 times the length of procoxae. Mentum subhexagonal, with lateral margins angulate at mid-length, anterior margin straight (Fig. 18a).

Elytra 1.15–1.18 mm long, 1.38–1.43 times as long as wide; 2.25–2.30 times as long as and 1.26–1.30 times as wide as pronotum; widest at basal fourth then continuously strongly converging to rounded apex. Punctation composed of small setiferous punctures and widely spaced, slightly larger, shallow foveate punctures (Fig. 18e).

Legs moderately long. Femora very narrow at base, then strongly widened at apical half. Tibiae moderately narrow, straight sided, continuously widening to apex. Metatibia very narrow, straight, continuous widening apically, 0.35–0.37 times as long as elytra; metatarsus 0.66 times as long as metatibia.

Abdomen with ventrite 1 slightly shorter than metaventrite and almost as long as three following ventrites combined; postcoxal lines on ventrite 1 shallow, nearly reaching 2/5 length of ventrite, irregularly rounded.

Male genital segment with sternite emarginate apically, and strongly acuminate at its base. Tegmen shorter than median lobe, narrower at basal third, then widening towards apex, with long setae on apical margin. Median lobe cylindrical, weakly curved in lateral view, narrowing near moderately acuminate apex (Fig. 10d–f).

Female genitalia with moderately broad coxites and with styli vestigial (Fig. 10g); spermatheca not studied.

**Figure 16.**
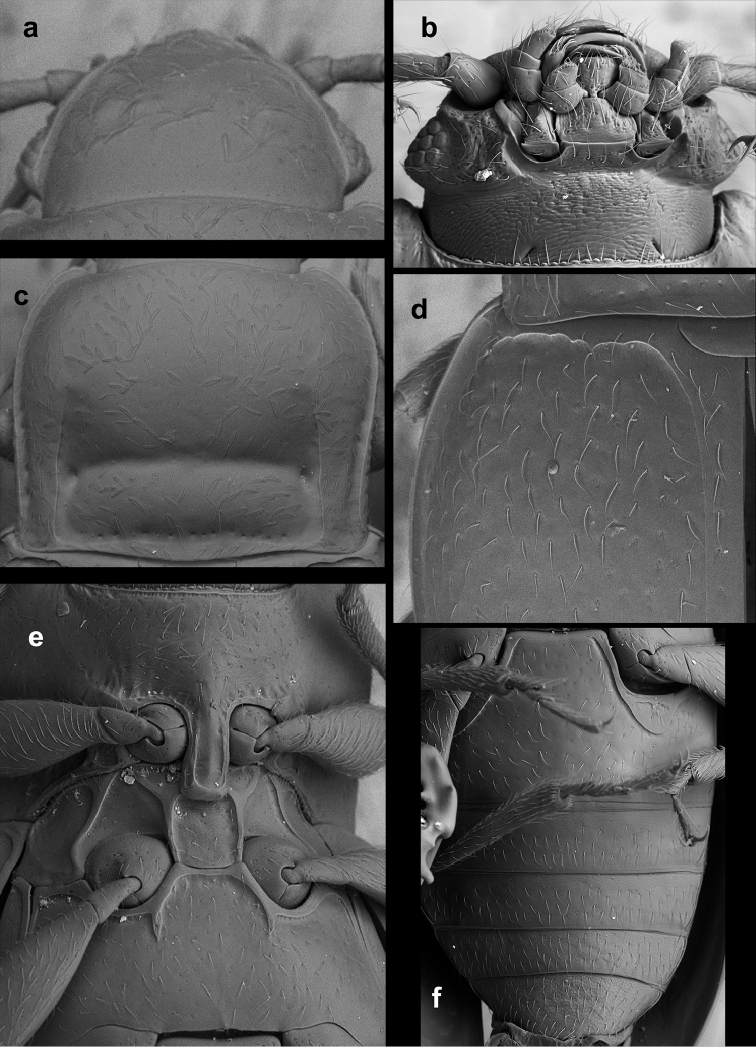
*Rueckeria inecol* sp. n. **a** dorsal view of head **b** ventral view of head **c** pronotum **d** antero-lateral corner of elytron **e** pro-, meso-, and metaventrite **f** abdominal ventrites.

**Figure 17.**
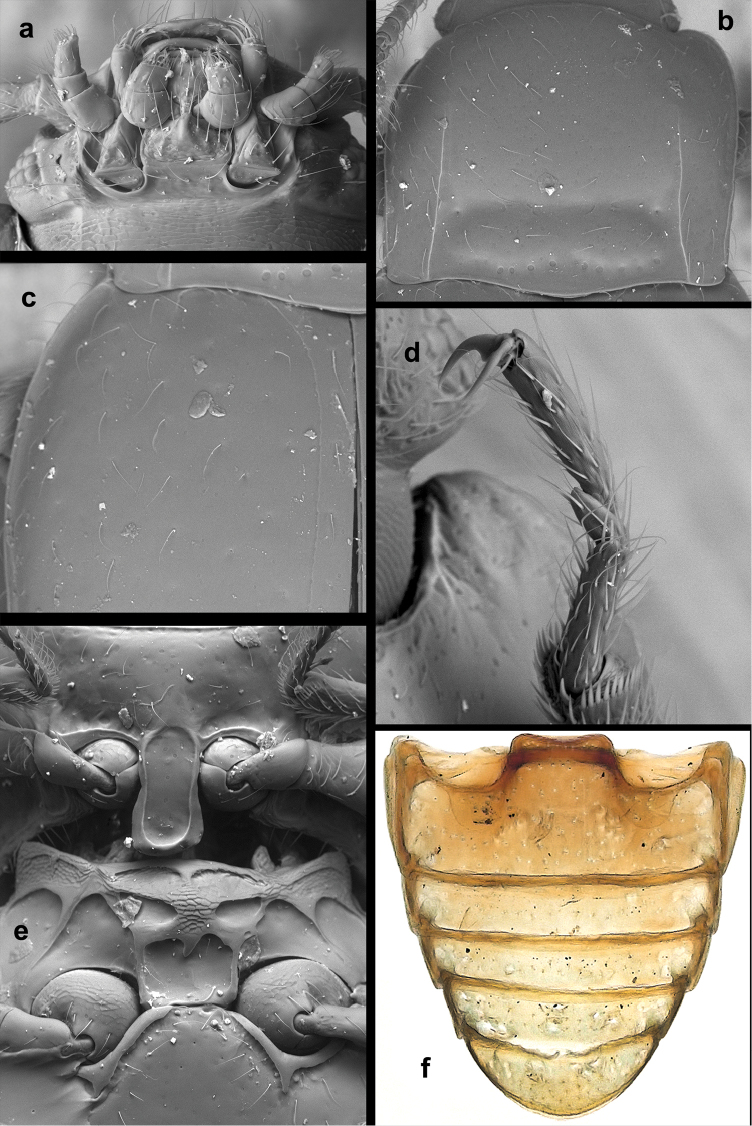
*Rueckeria nigrileonis* sp. n. **a** ventral view of head **b** pronotum **c** antero-lateral corner of elytron **d** protarsus **e** pro-, meso- and metaventrite **f** abdominal ventrites.

**Figure 18.**
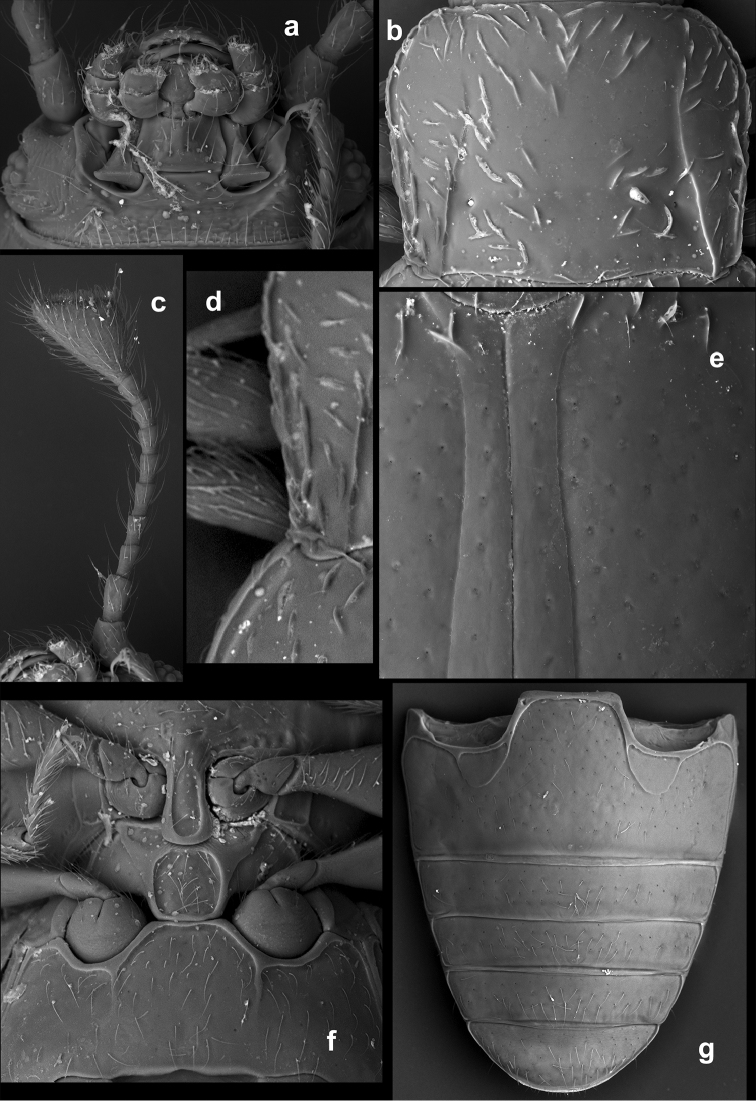
*Rueckeria ocelotl* sp. n. **a** ventral view of head **b** pronotum **c** antenna **d** posterolateral corner of pronotum and antero-lateral corner of elytron **e** details of elytral surface in anterior half **f** pro-, meso- and metaventrite **g** abdominal ventrites.

**Figure 19.**
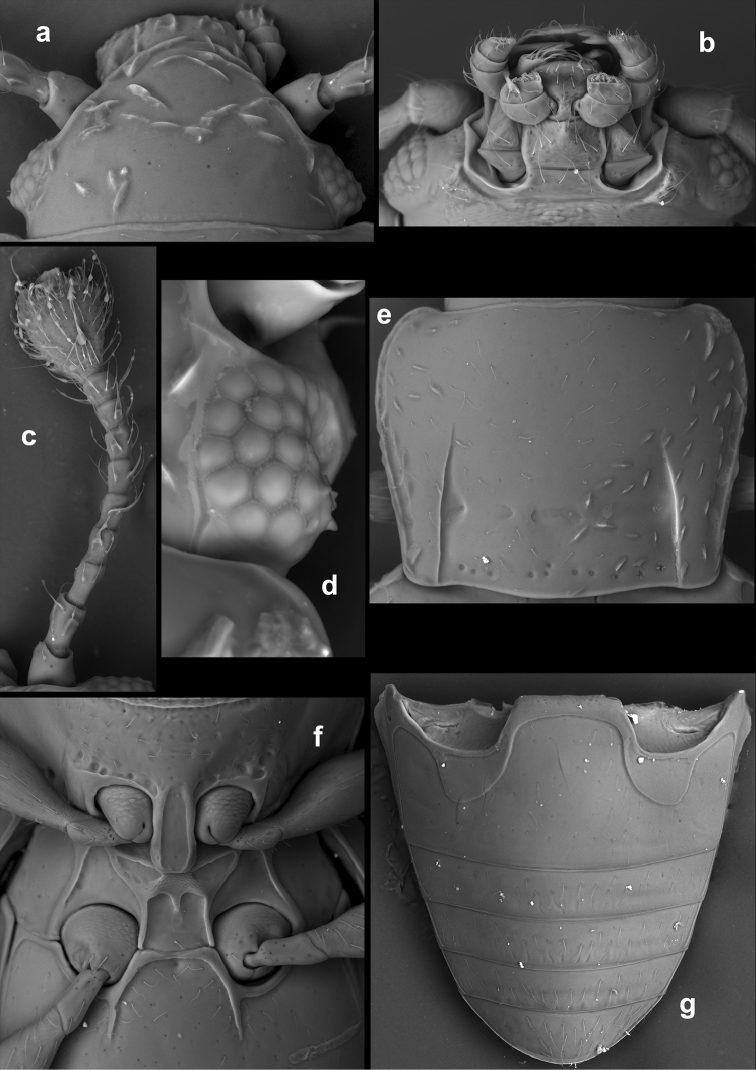
*Rueckeria puma* sp. n. **a** dorsal view of head **b** ventral view of head **c** antenna **d** latero-dorsal view of eye **e** pronotum **f** pro-, meso- and metaventrite **g** abdominal ventrites.

**Figure 20.**
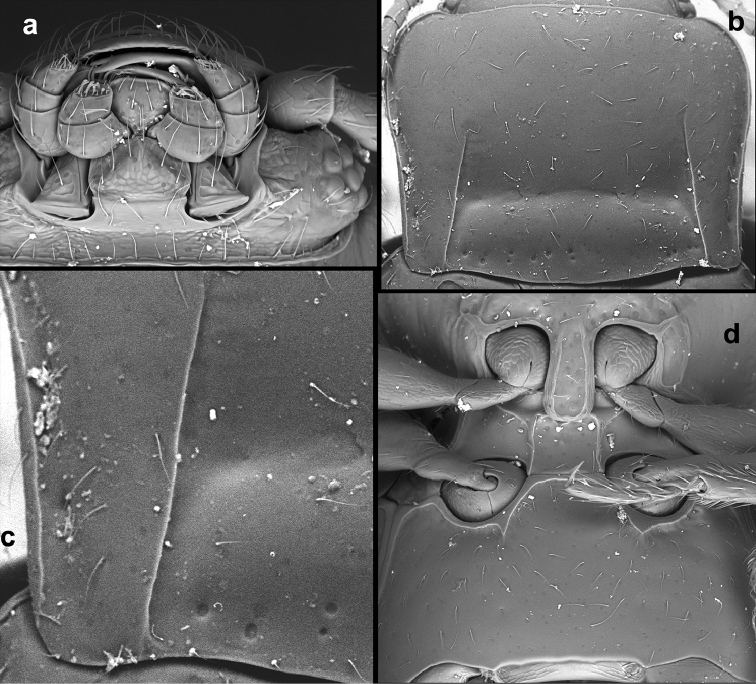
*Rueckeria skelleyi* sp. n. **a** ventral view of head **b** pronotum **c** posterolateral corner of pronotum **d** pro-, meso- and metaventrite.

**Figure 21.**
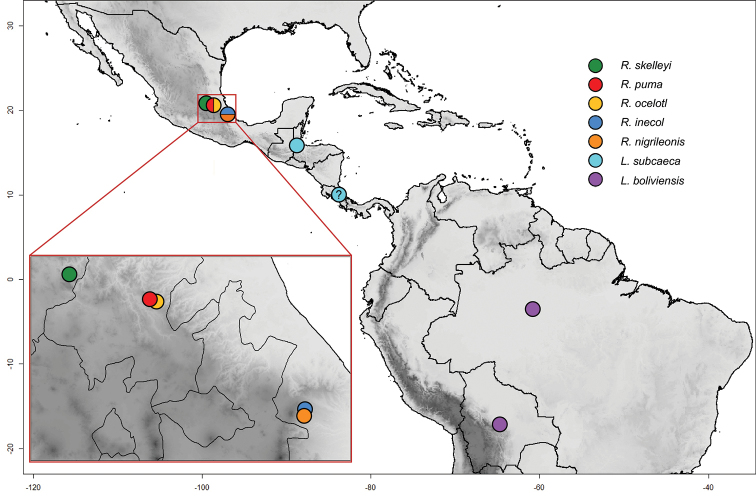
*Lycoperdinella* and *Rueckeria* species distribution map. (**green**) *R. skelleyi* sp. n. (**red**) *R. puma* sp. n. (**yellow**) *R. ocelotl* sp. n. (**dark blue**) *R. inecol* sp. n. (**orange**) *R. nigrileonis* sp. n. (**pale blue**) *L. subcaeca* Champion (**purple**) *L. boliviensis* sp. n.

##### Type material.

**Holotype**, **male, MEXICO**: “MEXICO: Hidalgo La Mojonera 4.8 km SE Zacualtipan, upper part of *Fagus* forest; 20°37.9'N 98°37.0'W; 2010 m; 13-16.ix.2016; Arriaga, Cortés, Fikáček & Seidel lgt. 2016-MX22 / sifting of large accumulations of leaf litter in relictual *Fagus* forest with intermixed Magnolia and tree ferns, with sparse to dense understory and many (partly rotten) fungi and logs” (NMPC); Paratypes, same data: (1 male: CZUG; 1 female: MIZ).

### Key to species of *Rueckeria*

**Table:** 

1	Color black with yellow antennae and legs (Figs 9a–c, 11a–c)	**2**
–	Color reddish brown with paler antenna and legs (Figs 8a–c, 10a–c, 12a–c)	**3**
2	Pronotum more strongly narrowed at base (1.20 times wider at widest part than at base) (Fig. 19b); aedeagus with apex of tegmen acuminate (Fig. 11d–f); coxites with large styli (Fig. 11g)	***R. puma* sp. n**
–	Pronotum less strongly narrowed at base (1.05 times wider at widest part than at base) (Fig. 17b); aedeagus with apex of tegmen weakly rounded (Fig. 9d–f); coxites with styli vestigial (Fig. 9g)	***R. nigrileonis* sp. n**
3	Pronotum with margins weakly crenulate (Fig. 18d); mentum subhexagonal with lateral margins angulate at mid length (Fig. 18); tegmen distinctly shorter than median lobe, widened apically (Fig. 10d–f)	***R. ocelotl* sp. n**
–	Pronotum with margins smooth (Figs 16c, 20b, c); mentum subrectangular with lateral margins weakly rounded (Figs 16b, 20a); tegmen at least as long as median lobe, subparallel-sided (Figs 8d–f, 12d–f)	**4**
4	Pronotum without perforated basal lateral pores, with just feeble and slightly depressed foveae (Fig. 20b, c); abdominal postcoxal lines reaching about half length of ventrite (Fig. 12g); tegmen much larger than median lobe, without tegminal strut; median lobe of aedeagus strongly acuminate apically (Fig. 12d–f)	***R. skelleyi* sp. n**
–	Pronotum with perforated basal lateral pores (Fig. 16d); abdominal postcoxal lines reaching about 1/3 of ventrite length (Fig. 16f); tegmen about as long as median lobe, with tegminal strut present; median lobe of aedeagus continuously widened apically (Fig. 8d–f)	***R. inecol* sp. n.**

## Discussion

The findings presented in this work reveal that the merophysiine fauna in the Neotropics is very poorly known. As a result, its diversity has been highly underestimated, while some monotypic genera considered as part of the subfamily were never re-examined and are most likely misclassified. This situation is caused in part to the lack of interest in studying small-sized beetles associated with environments such as forest leaf litter. Recent effort on studying the leaf-litter dwelling beetles from the cloud forests of Mexico revealed an unexpected diversity of merophysiine representatives that did not completely fit in the current generic concepts available. For those species a new genus, *Rueckeria*, is established here. Its members are evidently closely related to *Lycoperdinella*, a genus that was previously considered as monotypic. The distribution of *Lycoperdinella* is greatly expanded, from Guatemala and Costa Rica where it was previously recorded to Bolivia and Brazil. In contrast to *Lycoperdinella*, where species seem to be broadly distributed, representatives of *Rueckeria* probably occur locally and have higher speciation-rate caused by the lack of flying ability. So far *Rueckeria* is known only from Mexican biogeographical provinces: Trans-Mexican Volcanic Belt and Sierra Madre Oriental. However, it cannot be stated that its distribution is restricted to these provinces. Additional collecting efforts will likely result in new discoveries at the specific level and could provide more DNA-grade specimens that will help us testing the monophyly of the genera. In parallel, the type specimens of the enigmatic genera *Colovocerida* Belon, *Pseudevolocera* Champion, and *Pseudoparamecus* Brèthes, must be studied in order to elucidate their systematic position.

## Supplementary Material

XML Treatment for Lycoperdinella

XML Treatment for Lycoperdinella subcaeca

XML Treatment for Lycoperdinella boliviensis

XML Treatment for Rueckeria

XML Treatment for Rueckeria inecol

XML Treatment for Rueckeria puma

XML Treatment for Rueckeria nigrileonis

XML Treatment for Rueckeria skelleyi

XML Treatment for Rueckeria ocelotl
